# Therapeutic Effects of Green Tea Polyphenol (‒)-Epigallocatechin-3-Gallate (EGCG) in Relation to Molecular Pathways Controlling Inflammation, Oxidative Stress, and Apoptosis

**DOI:** 10.3390/ijms24010340

**Published:** 2022-12-25

**Authors:** Daniela Mokra, Marta Joskova, Juraj Mokry

**Affiliations:** 1 Department of Physiology, Jessenius Faculty of Medicine in Martin, Comenius University in Bratislava, SK-03601 Martin, Slovakia; 2 Department of Pharmacology, Jessenius Faculty of Medicine in Martin, Comenius University in Bratislava, SK-03601 Martin, Slovakia

**Keywords:** epigallocatechin-3-gallate, green tea, inflammation, oxidative stress, apoptosis, cancer, neurological, cardiovascular, respiratory, metabolic

## Abstract

(‒)-Epigallocatechin-3-gallate (EGCG) is the most abundant polyphenol in green tea. Thanks to multiple interactions with cell surface receptors, intracellular signaling pathways, and nuclear transcription factors, EGCG possesses a wide variety of anti-inflammatory, antioxidant, antifibrotic, anti-remodelation, and tissue-protective properties which may be useful in the treatment of various diseases, particularly in cancer, and neurological, cardiovascular, respiratory, and metabolic disorders. This article reviews current information on the biological effects of EGCG in the above-mentioned disorders in relation to molecular pathways controlling inflammation, oxidative stress, and cell apoptosis.

## 1. Introduction

The pathophysiology of many serious diseases is linked with inflammation and inflammation-induced oxidative stress. Excessive accumulation and activation of inflammatory cells leads to overproduction of a variety of biologically active substances including pro-inflammatory cytokines and reactive oxygen (ROS) and nitrogen species (RNS). Dysregulation of inflammation and oxidant/antioxidant disbalance may result in chronic tissue damage and organ dysfunction. Increased markers of inflammation and oxidative stress have been recently demonstrated in many distinct disorders including cancer [1,2], cardiovascular diseases [3,4], metabolic disorders including diabetes [5,6], chronic kidney disease [7,8], and neurodegenerative disorders [9,10]. In addition, inflammation with oxidative stress plays an important role in respiratory disorders such as acute lung injury including COVID-19 [11,12], chronic obstructive pulmonary disease (COPD) [13,14], bronchial asthma [15,16], pulmonary fibrosis [17,18], sarcoidosis [19,20], or silicosis [21,22].

Understanding the fundamental role of inflammation and inflammation-related oxidative stress in the onset and progression of the above-mentioned diseases has led to the successful use of various antioxidants including those of natural origin in their treatment [21,23,24,25,26,27,28,29,30,31,32,33,34,35,36,37]. Among the bioactive compounds present in various plants or fruits, a wide group of polyphenols should be considered [38]. Of the polyphenols of the green tea plant (*Camellia sinensis*), one that is exceptional is epigallocatechin-3-gallate (EGCG), which has shown a broad spectrum of anticancer, anti-inflammatory, antioxidant, vasoprotective, and antifibrotic actions [39,40,41,42,43].

This article summarizes current information on the anti-inflammatory and antioxidant effects of EGCG in relation to several selected diseases and critically discusses the effectiveness of EGCG administration in the preclinical conditions and in clinical studies. For this review, articles in English language from the PubMed database were used.

## 2. Epigallocatechin-Gallate (EGCG)

### 2.1. Green Tea Catechins

**Figure 1:**
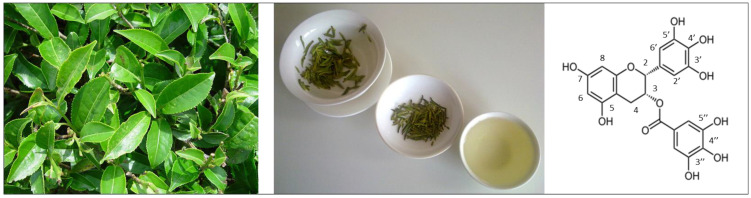
The green tea (*Camelia sinensis*) plant [47], appearance of green tea at three stages—the infused leaves, the dry leaves, and the green tea infusion [48]—and chemical structure of EGCG [49].

Green tea is rich in many polyphenols, i.e., flavanols, flavandiols, flavonoids, and phenolic acids. Major components of green tea polyphenols are flavanols (or catechins), of which the most abundant are (-)-epigallocatechin-3-gallate (EGCG), (-)-epicatechin (EC), (-)-epicatechin-3-gallate (ECG), and (-)-epigallocatechin (EGC) [44]. EGCG forms more than 50 % of all green tea catechins representing about 16.5 % of the water-extractable fraction of tea [45]. A cup of brewed tea contains about 200–300 mg of EGCG [39,46] (Figure 1).

### 2.2. Pharmacological Properties of EGCG

**Figure 2:**
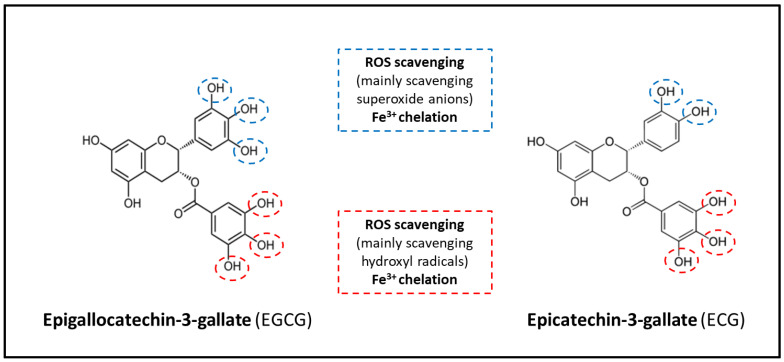
The biochemical structures of epigallocatechin-3-gallate (EGCG) and epicatechin-3-gallate (ECG) in relation to some of their antioxidant actions [51,52,54,55].

Plasma concentration of catechins reaches a peak value between 1–4 h after oral ingestion of green tea or catechin supplements and returns back to its baseline value within 24 h [50]. However, the individual polyphenols of green tea show rather big differences in their pharmacokinetics and bioavailability and thereby also in their biological effects [39,40]. It is presumed that these differences may be partially related to structural characteristics of the molecules. EGCG and ECG, the two most potent green tea catechins, contain the galloyl moiety which may be responsible for the stronger biological effects of these two substances [40,51,52] (Figure 2). In addition, the structural differences may be responsible for differences in elimination half-time, as demonstrated in the rapidly elevated plasma levels of EGC with a short elimination half-time of 1.7 h, while EGCG plasma concentration increased slowly but persisted longer (with an elimination half-time of 3.9 h) [53]. Besides the presence of the galloyl moiety esterified at carbon 3 on the C ring, the presence of hydroxyl groups at carbons 3’, 4’, and 5’ on the B ring of EGCG molecule likely also contributes to the superior antioxidant activity of EGCG in comparison to other catechins [52,54,55] (Figure 1 and Figure 2).

Moreover, the biological effects of EGCG depend on the plasma concentration [40,56]. Low or moderate concentrations of EGCG (with plasma levels of ≤10 µM) may exert mainly an antioxidant action mediated by EGCG-induced production of low amounts of ROS necessary for the stimulation of signal transduction pathways promoting cell protection [57,58]. However, high concentrations of EGCG (>10 μM) show predominantly a prooxidant action while the direct prooxidant effects of EGCG result from its autooxidation, leading to the production of hydrogen peroxide. Indirect prooxidant effects are related to the generation of more potent ROS including hydroxyl radicals because of reducing Fe (III) to Fe (II) [59,60]. The EGCG-induced generation of ROS enhancing autophagy and cell death [61] may be utilized as an apoptosis-enhancing action in the treatment of tumors [56].

The efficacy of EGCG is additionally influenced by its conversion to dimer or multimer as well as by its modification to glucuronated and/or methylated forms. This causes its low bioavailability after oral delivery [62]. The enzymatic transformation of orally taken EGCG is already initiated by saliva where the hydrolysis of EGCG by esterases occurs [55]. The process continues in the intestine where EGCG is passively transported into the intestinal cells exerting higher hydrophobicity of EGCG compared to other catechins [62]. The metabolic changes continue in the liver where O-methylated and/or glucuronated conjugates originate as the result of glucuronidation and sulfation of the hydroxyl groups and O-methylation of the catechol groups. Since the mentioned conjugates have similar biological activity as free EGCG, it is supposed that the rapid effects of EGCG may be attributable to the direct cellular action of EGCG, while the chronic effects are likely related to the action of EGCG metabolites [40,63,64]. In addition, EGCG undergoes two other processes, autooxidation and epimerization. In autooxidation, EGCG loses hydrogen atoms that lead to the production of potentially deleterious substances, such as semiquinone radical intermediates, superoxide, and quinone-oxidized products [65]. In epimerization (i.e., reversion of the stereochemistry of the bond that bridges the B- and C-rings) due to the brewing of tea leaves or after oral intake, the majority of EGCG is converted to (-)-gallocatechin gallate (GCG) which has similar properties as the cis-form of EGCG, and no toxic by-products are generated [66].

## 3. Mechanisms of Action of EGCG

EGCG exerts a wide spectrum of actions mediated via interactions with various cell surface receptors, intracellular signaling pathways, and transcription factors in the nucleus [40]. A list of the most important actions of EGCG is provided in Table 1.

### 3.1. Interactions with the Cell Surface Receptors

EGCG can bind to several surface receptors initiating cell signaling pathways and thereby can regulate their activities [40,67].

EGCG, but not other tea catechins, exclusively binds to the *67-kDa laminin receptor* (67LR) that likely represents the essential cell surface receptor for the anti-tumor effect of EGCG [68,69]. It is presumed that EGCG activates the 67LR receptor, leading to subsequent activation of the apoptotic signaling Akt/eNOS/NO/cGMP/PKCδ pathway, whereas the upregulation of cyclic guanosine monophosphate (cGMP) (e.g., by phosphodiesterase-5 inhibitor vardenafil) acts as a rate-determining process of 67LR-dependent apoptosis [70,71,72].

In addition, EGCG inhibits the *toll-like receptor (TLR)4* signaling through the 67LR-dependent mechanism and thereby exerts anti-inflammatory action [73,74]. Activation of TLR4 leads to the activation of important pathways regulating inflammation and apoptosis such as the nuclear factor (NF)-κB, activator protein (AP)-1 through activating mitogen-activated protein kinases (MAPK), or interferon regulatory factor (IRF)3 [75]. Thereby, EGCG may interact with the mentioned pathways resulting in anti-tumor, antioxidative, anti-inflammatory, neuroprotective, and other effects as demonstrated in numerous studies [76,77,78,79,80]. Moreover, the EGCG-mediated decrease in TLR4 activity attenuated inflammation and improved insulin signaling in adipose tissue [81]. Similarly, EGCG alleviated hepatic insulin resistance and improved obesity-associated subacute hepatic inflammation in a rat model of nonalcoholic fatty liver disease through the TLR4 signaling pathway [82].

EGCG also influences the *cell surface growth factor receptors*, mainly receptor tyrosine kinases, which participate in many processes including cell proliferation, survival, and angiogenesis [40]. For instance, EGCG inhibited a platelet-derived growth factor receptor (PDGFR)-induced mitogenesis of vascular smooth muscle cells [83]. Inhibition of the epidermal growth factor receptor (EGFR) by EGCG resulted in potent anti-tumor effects as demonstrated by the inhibition of cell proliferation and migration in non-small cell lung cancer cells [84], reduced colorectal cancer cell growth [85,86], or reduced invasion of breast cancer cells [87]. Another cell surface receptor, the vascular endothelial growth factor receptor (VEGFR), stimulates angiogenesis and increases the growth of tumorous and atherosclerotic plaques [88]. EGCG inhibited VEGFR and thereby partially reduced the growth of colorectal cancer cells [89] or hepatocellular carcinoma cells [90]. Similarly, EGCG caused the inhibition of an insulin-like growth factor (IGFR)-1 which is involved in the development of hepatocellular carcinoma, colon carcinoma, pancreatic carcinoma, and other cancers [91,92,93]. 

**Table 1:** Targets and major biological actions of EGCG.

Targets	Modulation of EGCG	Biological Effects of EGCG
*Cell surface receptors*		
67LR	activation	induction of cancer cells apoptosis [70,94], anti-inflammatory action [73,74]
TLR4	inhibition	anti-inflammatory action [73,74], neuroprotection [78], alleviation of insulin resistance [82]
PDGFR	inhibition	inhibition of mitogenesis of vascular smooth muscle cells [83]
EGFR	inhibition	inhibition of cell proliferation, migration and invasion in various types of tumor cells [84,86,87]
VEGFR	inhibition	inhibited angiogenesis, suppressed growth of cancer [89,90]
IGFR	inhibition	suppression of growth of various cancers [91,92,93]
*Intracellular signaling pathways*		
Cytosolic Ca^2+^	elevation	various biological actions including vasodilation and cardioprotection [95,96]
cAMP	elevation	inhibition of platalet aggregation [97], vasodilation [95]
cGMP	elevation	vasodilation and cardioprotection [95], anti-tumor action [70]
MAPK	inhibition/activation	anti-inflammatory action [98,99], anti-tumor action [100,101], neuroprotection [102]
COX-2	inhibition	anti-tumor action [103,104], anti-inflammatory action [105], neuroprotection [106]
AMPK	activation	induction of cancer cell apoptosis [107,108], hepatic autophagy/promotion of lipid metabolism [109], anti-inflammatory action [110], neuroprotection [111]
PI3K/Akt/eNOS	inhibition/activation	attenuation of brain vasogenic edema [112], anti-inflammatory action [113], decreased neuronal and endothelial apoptosis [114,115]
*Nuclear transcription factors*		
NF-κB	inhibition	anti-inflammatory [98,116] and anti-oxidant action [117], inhibited proliferation of cancer [80,118], neuroprotection [78,119]
AP-1	inhibition	anti-inflammatory action [120], attenuation of myocardial ischemia-reperfusion damage [121], inhibition of cancer cell growth [122]
Nrf2/HO-1	activation	anti-oxidant and anti-inflammatory action [123,124], anti-cancer action [125,126], cardio- and vasoprotection [43,127], neuroprotection [112,128]
STAT1	inhibition	cardioprotective action [129], anti-tumor action [130,131], anti-inflammatory action [132,133], prevention of vascular remodeling [134]
STAT3	inhibition	anti-inflammatory action [135], anti-tumor action [131,136]

Abbreviations: AMPK: adenosine monophosphate-dependent kinase, AP-1: activator protein 1, Ca^2+^: calcium ions, cAMP: cyclic adenosine monophosphate, cGMP: cyclic guanosine monophosphate, COX-2: cyclooxygenase-2, EGCG: epigallocatechin-gallate, EGFR: epidermal growth factor receptor, HO-1: heme oxygenase-1, IGFR: insulin-like growth factor receptor, 67LR: 67-kDa laminin receptor, MAPK: mitogen-activated protein kinase, NF-κB: nuclear factor kappa-B, Nrf2: nuclear factor erythroid-derived 2-like 2, PDGFR: platelet-derived growth factor receptor, PI3K/Akt/eNOS: phosphoinositide-3-kinase/protein kinase B/endothelial nitric oxide synthase, STAT1/3: signal transducer and activator of transcription 1/3, TLR4: toll-like receptor 4, VEGFR: vascular endothelial growth factor receptor.

### 3.2. Interactions with Intracellular Signaling Pathways

EGCG enhances the production of *signaling molecules* such as ROS, calcium ions (Ca^2+^), cyclic adenosine monophosphate (cAMP), or cGMP. They serve as second messengers for several downstream signaling pathways [40]. For instance, EGCG-induced low concentrations of ROS contribute to cell apoptosis [137,138]. The EGCG-evoked increase in cytosolic Ca^2+^ is essential for nitric oxide (NO)-mediated vasodilation and cardioprotection [95,96]. EGCG increases the levels of cAMP in platelets and may thereby inhibit collagen-induced platelet aggregation [97]. In endothelial cells, it may result in vasodilation [95,139]. EGCG increases cGMP levels via the cell surface receptor 67LR that stimulates the Akt/eNOS pathway and leads to vasodilation and improved cardiovascular function [95]. However, the inhibition of phosphodiesterase (PDE)-5 by vardenafil led to sustained elevation of cGMP and caused significant apoptosis suggesting promising anti-tumor therapy with a combination of EGCG and the PDE-5 inhibitor [40,70].

*The MAPK pathway* is one of the most important intracellular pathways influenced by EGCG. MAPK is involved in regulation of the cellular response to a wide spectrum of stimuli including mitogens and pro-inflammatory cytokines [140]. The family of MAPK is divided into three modules. The extracellular signal-regulated kinases (ERK)1/2 module is stimulated by growth factors and mitogens and regulates cell proliferation and differentiation. The second one, the c-Jun N-terminal kinase (JNK)/p38 module is activated, e.g., by oxidative stress and pro-inflammatory cytokines, and contributes to the control of cell differentiation, grow/cell cycle arrest, cell apoptosis, and inflammation. The third one, the ERK5 module is triggered by morphogenic clues and results in endothelial lumen formation [141]. The effect of EGCG on MAPK was demonstrated in numerous studies. For instance, EGCG inhibited a biosynthesis of aflatoxin B1 and alleviated the associated oxidative stress via downregulation of the MAPK signaling pathway [142]. EGCG induced the expression of β-defensin 3, an antiviral peptide produced by epithelial cells, as well as expression through upregulation of the p38 MAPK, ERK, and JNK signaling pathways that resulted in the inhibited replication of influenza A virus H1N1 [143]. EGCG attenuated in vitro hemolysis induced by α-hemolysin, a product of *Staphylococcus aureus*. In addition, EGCG decreased α-hemolysin-induced overproduction of ROS and reduced the expression of NLRP3 inflammasome and inflammasome-related generation of caspase-1, IL-1β and IL-18 in mice. This was associated with decreased activation of the MAPK signaling pathway, confirming ROS and MAPK as major activators of NLRP3 inflammasome [99]. In cardiomyocytes exposed to cigarette smoke, EGCG attenuated oxidative stress and prevented antioxidant depletion, reduced production of IL-8 and inhibited cell apoptosis. This was linked with inhibition of ERK1/2, p38 MAPK, and NF-κB pathways [98]. In another study, EGCG demonstrated its neuroprotective potential as it lowered ROS levels and inhibited apoptosis and enhanced expression of the brain-derived neurotrophic factor through downregulation of MAPK and other downstream pathways [102]. Some anti-tumor effects of EGCG were demonstrated via suppression of MAPK [144,145] or, more frequently, via upregulation of MAPK that was linked to the induction of apoptosis and reduced angiogenesis [85,100,101].

*Cyclooxygenase (COX)*, also known as prostaglandin-endoperoxide synthase, is an enzyme responsible for the formation of prostanoids from arachidonic acid, including thromboxane and prostaglandins such as prostacyclin [146]. Increased expression of COX-2 has been implicated in many pathologic conditions, including cancer and inflammation. Treatment with EGCG inhibited COX-2 without affecting COX-1 expression at both the mRNA and protein levels in human prostate carcinoma cells [104]. EGCG induced apoptosis of colon cancer cells and decreased the expression of inducible NO synthase (iNOS) and COX-2 and prostaglandin E(2) levels. However, it activated 5’ adenosine monophosphate-activated kinase (AMPK), responsible for the modulation of gene expression of COX-2 [147]. The decreased expression of COX-2 associated with inhibited cancer cell migration and invasion after EGCG treatment was demonstrated in various types of cancers [103,148,149,150]. Similar effect of EGCG on COX-2 activity was found in IL-1β-induced inflammatory changes in chondrocytes [151] as well as in lipopolysaccharide-stimulated macrophages [105]. The downregulation of COX-2 by EGCG may also decrease neuroinflammation and thereby contribute to neuroprotection [106].

The *AMPK* is an enzyme which plays an important role in maintaining cell energy homeostasis, regulates cell cycle, and activates autophagy and antioxidant defense [152]. In lipopolysaccharide (LPS)/interferon (IFN)-γ-stimulated mesangial cells, EGCG activated AMPK and blocked iNOS and thereby attenuated inflammation [110]. The neuroprotection effect of EGCG through the activation of AMPK was demonstrated both in an in vivo model of traumatic brain injury where EGCG ameliorated neurological impairment, including spatial learning and memory [111], as well as in in vitro measurements on microglia cells [106]. The pro-apoptotic effects of EGCG mediated via AMPK activation and COX-2 inhibition were demonstrated in colon cancer cells [107,147] and human hepatoma cells [108]. In addition, EGCG increased hepatic autophagy by promoting the formation of autophagosomes, increasing lysosomal acidification, and stimulating autophagic flux in hepatic cells and in vivo. This lipid clearance effect can be attributable to increased phosphorylation of AMPK, one of the major regulators of autophagy. By this action, EGCG may promote lipid metabolism and induce hepatic autophagy, and thereby contribute to reduced hepatosteatosis [109].

*Phosphatidylinositol-3-kinase (PI3K)-protein kinase B (Akt) signaling pathway* is a signal transduction pathway promoting survival and growth in response to various extracellular signals such as hormones, growth factors, or components of extracellular matrix. PI3K-activated Akt regulates the function of many proteins involved in metabolism, apoptosis, and proliferation and PKB/Akt is active in various types of cancer. Activating eNOS, Akt contributes also to angiogenesis [153]. EGCG inhibited neovascularization and attenuated vasogenic edema following *status epilepticus* via downregulation of the PI3K/Akt/eNOS pathway [112]. EGCG inhibited inflammatory cell infiltration into the lungs of ovalbumin-challenged asthmatic mice, decreased levels of interleukins (IL)-4, IL-5 and transforming growth factor (TGF)-β1, and decreased epithelial-mesenchymal transition (EMT) via inhibiting the PI3K/Akt signaling pathway [113]. In immune-stimulated mesangial cells, EGCG effectively inhibited the immune-stimulated PI3K/Akt/mTOR pathway independently of AMPK, by decreasing phosphorylation of Akt [110]. In contrast, activation of the PI3K/Akt/eNOS pathway was likely responsible for the alleviation of endothelial dysfunction and apoptosis in high glucose-induced dysfunction of umbilical vein endothelial cells [115]. In another study, EGCG-induced upregulation of the PI3K/Akt/eNOS pathway resulted in neuroprotective effects, as demonstrated by lower neuronal degeneration and necrosis, lower neuronal apoptosis, and decreased oxidative stress [114].

### 3.3. Interactions with Nuclear Transcription Factors

Transcription factor *NF-κB* is activated by various stimuli such as oxidative stress, cytokines, bacterial or viral antigens, oxidized low-density lipoprotein, etc. It participates in the regulation of various genes that are important for cell responses, including inflammation, innate immunity, growth, and cell death [154]. In the cytoplasm, NF-κB is present in an inactive form through interaction with the inhibitor of κB (IκB), while the phosphorylation of IκB by IκB kinase causes ubiquitination and degradation of IκB. The subsequent releasing of NF-κB enables its translocation to the nucleus. Phosphorylation and activation of IκB kinase is controlled by an NF-κB-inducing kinase which represents a crosstalk between activation of the MAPK/ERK pathway, and the NF-κB-inducing kinase/IκB kinase/NF-κB pathway [46]. The positive effects of EGCG treatment related to the inhibition of NF-κB have been demonstrated in numerous studies. For instance, the neuroprotective effects of EGCG were found in hypoxic microglia cells where EGCG diminished production of ROS and IL-6 in the cells, decreased expression of the hypoxia-inducible factor, and inhibited inducible NO synthase and COX-2 via the inhibition of NF-κB [119]. However, additional studies confirmed the attenuated neuroinflammation and oxidative stress after treatment with EGCG [78,119,155]. In other studies, EGCG inhibiting NF-κB suppressed inflammation in endothelial cells [116,156] and in cardiomyocytes [98]. EGCG reduced cigarette-smoke-induced oxidative stress and attenuated the expression of pro-inflammatory genes in bronchial epithelial cells [117,157]. EGCG-induced inhibition of NF-κB resulted in anti-inflammatory effects in relation to carcinogenesis, as well [158]. For instance, EGCG inhibited NF-κB activity in human colon cancer cells [103], bladder cancer cells [118], or lung cancer cells [159] that resulted in inhibited cancer cell proliferation and migration.

*AP-1* is a transcription factor activated by growth factors, oncoproteins, tumor necrosis factor (TNF)α, IL-1, and others. AP-1 participates in the regulation of genes involved in apoptosis and proliferation and may enhance cell proliferation and high AP-1 activity may be associated with tumor progression of various types of cancer [160]. EGCG inhibited the AP-1 activity that resulted in diminished oncogen-induced cell transformation in epidermal cell lines [161] but also in prostate cancer cells [122] or colon cancer cells [103]. Through suppression of both AP-1 and MAPK, EGCG contributed to the prevention of overexpression of matrix metalloproteinases (MMPs), which are closely related to tumor cell invasion or alteration of the tissue [144,162,163,164]. Inhibiting both NF-κB and AP-1 EGCG attenuated myocardial ischemia-reperfusion damage in rats [121] or inflammatory changes in primary T cells [120] and in a murine model of colitis [165].

*Nuclear factor erythroid 2-related factor 2 (Nrf2)-signaling pathway* is also involved in the regulation of many biological processes and its dysregulation is associated with the pathogenesis of various diseases including cancer [166]. Basal levels of Nrf2 are usually low in unstressed cells due to KEAP1-mediated proteasomal degradation. However, in oxidative stress or metabolic alteration the electrophiles- and ROS-induced oxidation and modification of KEAP1 sensor cysteines lead to inhibition of KEAP1-mediated Nrf2 degradation and thereby Nrf2 accumulates in the nucleus and activates cytoprotective and metabolic genes [167,168]. Thus, Nrf2 acts as a master transcriptional regulator of antioxidant response element (ARE)-containing cytoprotective genes whose expression is induced in response to cell stress. The products of these genes create a network of cooperating enzymes involved in phase I (e.g., NAD(P)H quinone oxidoreductase (NQO)-1), phase II (e.g., glutathione-S-transferase and heme oxygenase (HO)-1), or phase III, which are responsible for detoxification reactions and the metabolic elimination of prooxidants [169,170]. In acute or chronic inflammation, the activation of inflammatory cells leads to increased production of electrophiles which react with cysteine residues of KEAP1 and, subsequently, Nrf2 activation reduces an inflammation-associated oxidative stress [168,171]. In vascular endothelium, EGCG requires p38 MAPK to elevate expression of Nrf-2 enhancing expression of HO-1 that results into increased HO-1 activity, providing anti-inflammatory actions of EGCG [172]. Nrf2 activation may reduce the risk of cancer by suppressing oxidative stress and tumor-promoting inflammation. However, increased Nrf2 activity in many cancers may originate either due to mutations that disrupt the negative control of Nrf2 activity or other factors, and Nrf2 activation can even be associated with poor prognosis [168]. EGCG may act as a potent Nrf2 activator [166,173,174], and its favorable effects in cancer [125,126] or in cardiovascular [43,127,175], neurodegenerative [112,128,176], respiratory [177,178], or other diseases [179,180,181] mediated via activation of Nrf2 have been published in numerous articles. 

A family of *signal transducer and activator of transcription (STAT) proteins* includes intracellular transcription factors mediating immunity, cellular proliferation, apoptosis, and differentiation. The STAT pathway is primarily activated by membrane-receptor-associated Janus kinases (JAK) after interaction with interleukins (IL-2 up to IL-7), granulocyte-macrophage colony stimulating factor, growth hormone, epidermal growth factor (EGF), platelet derived growth factor (PDGF), and IFN [182,183]. Activation of this pathway contributes to various inflammatory diseases and stimulated angiogenesis enhances the survival of tumors and immunosuppression. 

STAT1 is activated by interferons and growth hormone and subsequently converts these signals into gene expression of the molecules, such as iNOS, COX, vascular cell adhesion molecules (VCAM), and intercellular cell adhesion molecules (ICAM). They are involved in various inflammatory diseases including asthma, celiac disease, or psoriasis. However, STAT1 is elevated also in ischemia/reperfusion injury, diabetes, atherosclerosis, or unstable angina pectoris [184]. EGCG was identified as a potent inhibitor of STAT1 in IFN-γ elicited STAT1 activation in various cell lines suggesting the anti-inflammatory and anti-tumor action of EGCG [132]. EGCG pretreatment ameliorated lung edema, decreased histological signs of lung injury, lowered the production of pro-inflammatory cytokines TNFα and IL-1, and elevated levels of anti-inflammatory IL-10 in a rat model of seawater-aspiration-induced acute lung injury. In contrast, it also prevented an increase in TNFα and IL-1 and a decrease in IL-10 in rat alveolar macrophage cell lines [133]. In vascular endothelial cells, EGCG suppressed STAT1 pathway and IFN-γ-induced upregulation of P2X4-receptor mRNA [134]. P2X4 receptors of endothelial cells mediate the shear stress-induced calcium influx and production of NO and thereby regulate blood pressure and vascular remodeling [185]. However, upregulation of the P2X4 receptor results in an exaggeration of ATP-induced Ca^2+^ response and may contribute to vascular remodeling [134]. EGCG reduced STAT1 phosphorylation and protected cardiac myocytes from ischemia/reperfusion-induced apoptosis that was linked with enhanced hemodynamic recovery and ventricular function in the ischemic/reperfused rat heart [129]. Inhibiting STAT1, EGCG may also exert anti-tumor activity e.g., by a decrease of IFN-γ-induced expression of indoleamine 2,3-dioxygenase, which enhances progression of tumor cells [130].

STAT3 is activated by IL-6 and other cytokines suggesting its roles in the inflammatory response [182,183]. EGCG treatment suppressed the STAT3 pathway and thereby showed anti-inflammatory effects in various tissues, e.g., reduced TNFα-induced lung inflammation [135], mitigated retinal inflammation in an LPS-induced model of anterior uveitis [186], and decreased neuroinflammation and apoptosis of the hippocampus and thereby alleviated an anxiety-like behavior after myocardial infarction in rats [187]. However, aberrant activation of STAT3 was also found in solid and hematological cancers whereas T-cell-produced cytokines can promote STAT3 in cancer cells to impact tumorigenicity [136]. EGCG pretreatment suppressed both the STAT1 pathway activated by IL-6 and the STAT3 pathway activated by IFN-γ in cholangiocarcinoma cells [131]. In colorectal cancer cells, EGCG-induced downregulation of STAT3 inhibited cell proliferation because of induction of apoptosis and reduced cell migration in a dose-dependent manner [188]. Similar anti-tumor effects of EGCG were observed in other studies, e.g., in breast cancer cells [189], pancreatic cancer cells [190], or gastric cancer cells [191].

## 4. Therapeutic Effects of EGCG

**Figure 3:**
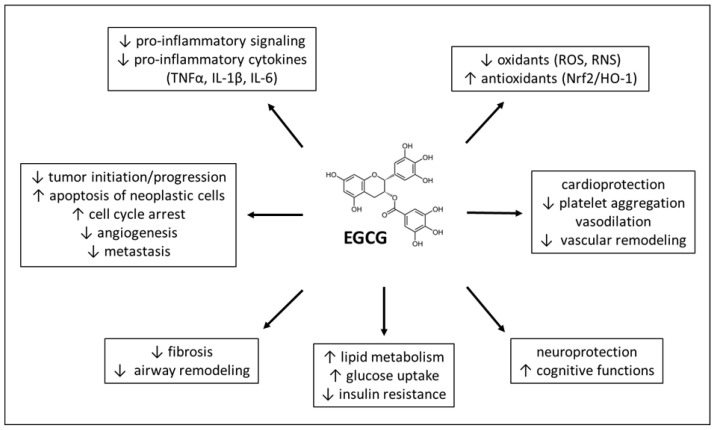
Pharmacological effects and therapeutic benefits of EGCG. Abbreviations: EGCG: epigallocatechin-3-gallate, HO-1: heme oxygenase, IL: interleukin, Nrf2: nuclear factor erythroid 2-related factor 2, RNS: reactive nitrogen species, ROS: reactive oxygen species, TNF: tumor necrosis factor, ↑: increase, ↓: decrease.

A variety of actions of EGCG (Figure 3) have been described particularly in relation to cancer [40,192,193]; however, an improvement associated with delivery of EGCG has also been observed in other disorders, such as neurological diseases including Parkinson’s and Alzheimer’s diseases [194,195], cardiovascular diseases [196,197], respiratory diseases [177,198], or metabolic diseases including obesity [179,199] and diabetes mellitus [200,201]. 

### 4.1. EGCG in Cancer

Anti-tumor action of EGCG is mediated via multiple pathways [40,67,202]. EGCG enhances gap junctional communication between the adjacent cells and thus protects the cells from tumor development, as tumor promoters inhibit gap junctional intercellular communication and isolate preneoplastic cells from the regulatory influence of surrounding cells, considered to be a key mechanism of tumor promotion [203]. 

In addition, the anti-tumor effects of EGCG are partially related to its wide anti-inflammatory and antioxidant effects, as EGCG may suppress chronic inflammatory processes resulting in cell transformation and hyperproliferation and initiation of carcinogenesis [41]. Cancer initiation and progression can be regulated by various proteins and signaling pathways which are also involved in inflammation and growth or death of cells. These are represented by transcription factors NF-κB, AP-1, STAT1/STAT3, etc., pro-apoptotic proteins including caspases or poly(ADP-ribose) polymerase (PARP), anti-apoptotic proteins including serine/threonine protein kinase Akt or B-cell lymphoma 2 regulator protein (Bcl-2), protein kinases, such as MAPK or JNK, cell cycle proteins, cell adhesion molecules, such as ICAM, COX-2, growth factor signaling pathways, and others [160,204].

EGCG-induced suppression of NF-κB results in both anti-inflammatory and anti-tumor effects [205,206] as NF-κB controls not only the synthesis of pro-inflammatory cytokines such as TNFα or IL-1β, but also contributes to the regulation of cell growth [46]. Similarly, EGCG via inhibition of MAPK and AP-1 pathways responsible for regulation of cell proliferation, differentiation, and death may influence inflammation and tumor progression [103,161,207]. A significant link between inflammation and cancer has been confirmed for NF-κB and STAT3. These two main pathways for inflammation are activated by the most important cancer risk factors, and a majority of gene products linked to inflammation, survival, proliferation, invasion, angiogenesis, and metastasis is controlled by NF-κB and STAT3. In contrast, suppression of NF-κB and STAT3 reduces the proliferation and invasion of tumors [208]. In addition, EGCG inhibits expression of COX-2 of which inappropriate activity was observed in a majority of premalignant and malignant conditions [104,160]. EGCG also reduces proteasome activity, which is responsible for the degradation of damaged or misfold proteins [209].

The additional anti-tumor effects of EGCG result from its potent antioxidant action [39]. The direct antioxidant action of EGCG is mediated via scavenging ROS and chelating free transition metals [210]. Indirect antioxidant effects may be related to (1) inhibition of redox-sensitive transcription factors, such as NF-κB or AP-1; (2) inhibition of pro-oxidant enzymes, such as iNOS or COX-2; and (3) induction of antioxidant enzymes, such as glutathione S-transferase or superoxide dismutase (SOD) [40,210,211]. In addition, EGCG induces the expression of Nrf2 and associated enzymes HO-1 and NQO-1, contributing to its antioxidant and anti-inflammatory effects [172]. The antioxidant actions of EGCG have been explained more in detail in our recent article [198]. There are also other biological effects of EGCG which are relevant in inflammation and/or cancer, such as strong inhibition of release of histamine and leukotriene B4, inhibition of Fas receptor and Na^+^/H^+^ exchanger, activation of silent information regulator 1, or increase in intracellular second messenger concentrations, such as Ca^2+^, cAMP, or cGMP [40]. In addition, EGCG blocks carcinogenesis via influencing other signaling pathways including PI3K/Akt [39,42].

Nevertheless, anti-cancer activity of EGCG is also associated with its ability to regulate a cell cycle and thereby to modulate the progression of a tumor. EGCG induces cell apoptosis and stimulates a cell growth arrest by interaction with proteins regulating the cell cycle, e.g., by direct inhibition of cyclin-dependent kinases [212,213]. EGCG activates effector caspases and suppresses oncogenic transcription factors and factors maintaining pluripotency [39]. EGCG also stimulates fragmentation of telomere via inhibition of telomerase activity, leading to cell apoptosis [214,215]. 

EGCG inhibits growth factors, e.g., EGFR and IGFR-1 and their signaling pathways, which suppresses a growth of tumor cells and metastasis [91,92,207]. EGCG also reduces cancer-related angiogenesis [216] by suppressing VEGFR gene expression and thus blocks tumor invasion and metastasis [217]. 

The anti-tumor action of EGCG has been confirmed in numerous in vitro experiments or in animal studies in which the interactions of EGCG with the above-mentioned cell surface receptors [70,84,87,89,90,91,92,93,94,96] and, subsequently, with intracellular signaling pathways [70,100,101,103,104,107,108] and nuclear transcription factors [80,103,118,122,125,126,130,131,136] have been shown (Table 1). The positive effects of the administration of EGCG in various types of tumors were demonstrated in several clinical studies [218,219,220,221,222,223,224,225], as well.

### 4.2. EGCG in Neurological Diseases

The benefits of EGCG and its metabolites in neurological disorders have been recently described in several excellent reviews [226,227,228]. Epidemiological studies in Japan, China, and Singapore have demonstrated a positive relation between drinking tea and improved cognitive functions or prevention of cognitive dysfunction [229,230,231,232,233,234]. These findings were supported by the results from numerous animal studies [235]. For instance, intragastric administration of EGCG for 60 days prevented cognitive deterioration in senescence-accelerated mice and decreased the accumulation of β-amyloid, which plays a fundamental role in Alzheimer’s disease [235]. In addition, EGCG reduces β-amyloid-induced cognitive dysfunction through modification of secretase activity via suppression of the ERK and NF-κB pathways [236]. In contrast, EGCG-induced extracellular degradation of the amyloid β-protein by increasing neprilysin secretion from astrocytes is mediated through activation of the ERK and PI3K pathways [237]. In other rodent models of Alzheimer’s disease, EGCG prevented a hyperphosphorylation of tau protein in hippocampus and reversed a decrease in synaptic proteins that resulted in lower impairments in memory and spatial learning [238,239].

EGCG treatment may also positively influence Parkinson’s disease as demonstrated in many epidemiological trials which showed that drinking tea may protect from Parkinson’s disease [240,241,242]. The neurological impairment in Parkinson’s disease is associated with a loss of dopaminergic neurons in *substantia nigra* and formation of cytoplasmic inclusions (Lewy bodies) from presynaptic protein α-synuclein which seem to be involved in oxidative stress and neuroinflammation [243]. In models of Parkinson’s disease, treatment with EGCG inhibited α-synuclein aggregation [244], prevented the decrease in dopamine [245,246], and reduced neuronal cell death that was associated with inhibition of iNOS [247]; however, influence by other mechanisms cannot be excluded [228]. 

### 4.3. EGCG in Cardiovascular Diseases

Tea consumption has appeared to be beneficial also in the prevention of atherosclerosis and coronary heart disease [43,196,197]. In the Ohsaki National Health Insurance Cohort Study carried out on 40,530 Japanese adults aged from 40 to 79 years, green tea consumption was inversely associated with mortality due to cardiovascular disease [206]. In a Norwegian study with 9856 men and 10,233 women without history of cardiovascular disease or diabetes aged from 35 to 49 years, drinking of green tea reduced the level of blood cholesterol and decreased blood pressure [248]. 

The key mechanisms responsible for EGCG-induced vasoprotection are represented by its antioxidant and anti-inflammatory effects. For instance, EGCG significantly decreased lipid peroxidation and increased levels of both non-enzymatic and enzymatic antioxidants in EGCG-treated rats compared with untreated animals within the atherosclerosis model [249]. The antioxidant effects of EGCG may be at least partially mediated by upregulating Nrf2/HO-1 via activation of p38 MAPK and ERK1/2 signaling pathways [124,166,211]. Moreover, EGCG suppresses inflammation in human coronary artery endothelial cells by inhibiting NF-κB, inhibits enhanced expression of adhesion molecules such as VCAM-1 and ICAM-1, and attenuates monocyte adhesion [116,250]. 

Polyphenols may additionally improve vascular function by other mechanisms. For instance, tea polyphenols decreased total cholesterol, low-density-lipoprotein (LDL)-cholesterol, plaque area/lumen area ratio, and enhanced gut microbiome, which reduces atherosclerotic plaque formation [251]. Moreover, treatment with EGCG enhanced endothelial function as indicated by improved brachial-artery-flow-mediated dilation [252]. Prevention of endothelial dysfunction and induction of vascular-endothelium-dependent vascular relaxation by polyphenols is likely mediated by redox regulation and NO production via activation of eNOS [197]. However, activation of eNOS by tea polyphenols is complex and depends on p38 MAPK and ligand-independent activation of estrogen receptor-α which leads to activation of the PI3K/Akt pathway and finally eNOS phosphorylation [139,253]. EGCG reduces production of endothelin-1 (ET-1) which acts as a potent vasoconstrictor but also increases a lipid biosynthesis and accelerates the progression of atherosclerosis [254,255]. EGCG inhibits VEGFR-2 signaling in endothelial cells and thus prevents angiogenesis and growth of atherosclerotic plaques induced by excessive concentrations of VEGF [256]. In addition, EGCG exhibited a potent antithrombotic activity and inhibition of platelet aggregation, which is mediated by multiple mechanisms including inhibition of cytoplasmic Ca^2+^ increase [257,258]. The above-mentioned studies demonstrate that, besides the ability of flavonoids to scavenge radicals, flavonoids activate specific signaling pathways in endothelial cells that improve multiple aspects of endothelial function [259].

### 4.4. EGCG in Respiratory Diseases

EGCG has been increasingly used also in the treatment of various acute and chronic respiratory diseases [177,198]. For instance, in TNFα-induced inflammation EGCG suppressed ICAM-1 expression, oxidative stress, MAPK and STAT3 activation, and reduced increases in eosinophil and neutrophil counts in the bronchoalveolar lavage fluid (BALF) [135]. In pulmonary inflammation caused by intratracheal LPS, EGCG alleviated lung injury and edema, decreased counts of inflammatory cells in the lung, decreased activities of myeloperoxidase (MPO) and proteinkinase Cα, lowered levels of TNFα, IL-1β, and IL-6 [260], and mitigated oxidative damage and enhanced lung regeneration [261]. In systemic inflammation induced by intraperitoneal LPS, EGCG pretreatment enhanced gas exchange, decreased lung injury, reduced MPO activity and expression of TNFα, IL-1β, and IL-6, alleviated expression of TLR4, and elevated expression of IκB-α, suggesting the relation of anti-inflammatory action of EGCG to suppressed activation of TLR4-dependent NF-κB signaling pathway [80]. 

The anti-bacterial properties of EGCG have been demonstrated in several animal models of pneumonia. For instance, EGCG reduced signs of lung injury and edema, decreased *Pseudomonas aeruginosa* load and virulence factors, decreased TNFα, IL-1β, IL-6, and IL-17 and elevated anti-inflammatory cytokines IL-4 and IL-10 [262]. Similarly, microencapsulated EGCG given for 6 weeks by pulmonary delivery led to resolution of inflammation in the *Mycobacterium tuberculosis*-infected lung by enhancing the autophagy and reduction in bacterial burden [263]. The potent antiviral activity of EGCG was confirmed in in vivo and in vitro models of influenza A [264].

EGCG has also shown its therapeutic potential in COVID-19. Via activation of the Nrf2 pathway, EGCG blocked infection with *severe acute respiratory syndrome coronavirus* 2 (SARS-CoV-2) by inhibiting the spike binding to angiotensin-converting enzyme 2 (ACE) receptor, a cell receptor for SARS-CoV-2 cell entry [265,266]. Moreover, EGCG mitigated a replication of SARS-CoV-2 through inhibition of the main protease (3CLpro) of the virus [267,268,269]. EGCG-suppressed SARS-CoV-2 replication may be also attributable to the decreased generation of ROS in mitochondria and lower oxidative burst linked with neutrophil extracellular traps (NETs) [270]. EGCG may also inhibit a life cycle of SARS-CoV-2 by suppression of endoplasmic reticulum-resident glucose-regulated protein (GRP)78 activity [271]. In addition, EGCG mitigated a cytokine storm in COVID-19 by downregulation of TLR4 and NF-κB and alleviated COVID-19-associated complications, such as sepsis, thrombosis, or lung fibrosis [178]. EGCG, directly or through suppressing STAT1 activation, reduces high mobility group box (HMGB)1, a redox-sensitive pro-inflammatory nuclear protein mediating sepsis [272,273]. Moreover, EGCG modulated the activity of platelets via inhibiting cytoplasmic Ca^2+^ elevation [257] and prevented thrombosis via decreasing tissue factors [274]. 

EGCG can also be effective in bronchial asthma. In ovalbumin-evoked models of asthma, EGCG decreased mucus production, expressions of p38 MAPK and matrix metalloproteinase (MMP)-9 [275], mitigated inflammatory cell infiltration, and inhibited TGF-β1 and PI3K/Akt signaling-pathway-induced EMT. This suggests the ability of EGCG to prevent airway remodeling [113,276]. In addition, EGCG demonstrated anti-inflammatory and antioxidant effects in a model of allergic asthma associated with obesity [277], as well as in models of asthma evoked by inhalation of toluene diisocyanate [278], fine particular matter [279], or house dust mite [280]. 

In cigarette-smoke-induced models of chronic obstructive pulmonary disease, EGCG via suppression of NF-κB decreased markers of oxidative stress and reversed activities of antioxidant enzymes, lowered neutrophil infiltration in the lung and markers of neutrophil-mediated inflammation, reduced secretion of mucus likely via inhibition of EGFR, and mitigated small airway remodeling by decreasing collagen deposition [117,281]. 

EGCG was also of benefit in the treatment of lung fibrotizing diseases which result from chronic activation of NF-κB and overproduction of pro-inflammatory cytokines and proteolytic enzymes, depletion of antioxidant system Nrf2, activation of growth factors, increased expression of fibrogenic and angiogenic factors leading to elevated MMPs, smooth muscle actin (SMA), collagen, etc. [282]. In bleomycin-induced models of lung fibrosis, EGCG treatment prevented a decrease in body weight, reduced markers of inflammation including levels of TNFα and IL-1β and activities of NF-κB and MPO, decreased markers of lipid peroxidation and increased levels of antioxidants enhancing Nrf2 activity, reduced lung edema, decreased content of hydroxyproline, a collagen breakdown product, and improved the histological picture of the lung [283,284,285] that was associated with the downregulation of MMP-2 and MMP-9, TGF-β1, and α-SMA [286]. Mitigation of TGF-β1 signaling and activation of MMP-dependent collagen I turnover by EGCG has been also confirmed in cultured lung slices from explants of patients with idiopathic pulmonary fibrosis [287]. EGCG demonstrated favorable effects on inflammatory and fibrotic changes in other animal models, e.g., in irradiation-induced fibrosis where anti-oxidant effects were in relation with activation of Nrf2 and associated antioxidant enzymes HO-1 and NQO-1 [123], or in cyclophosphamide- [288] and paraquat-induced induced models of pulmonary fibrosis [289].

In the lung silicosis, another therapeutic target for EGCG, oxidative stress and inflammation caused by persistence of inhaled silica particles in the lung can be alleviated by delivery of naked EGCG or the therapeutic effect of EGCG can even be enhanced by its encapsulation [290].

### 4.5. EGCG in Metabolic Diseases

EGCG also demonstrates favorable effects on the metabolism of lipids and associated obesity and metabolic syndrome [196,291,292]. In animal experiments, administration of EGCG decreased body weight, percent of body fat and visceral fat weight in high-fat-fed mice, alleviated insulin resistance, decreased triglycerides in the liver, and reduced plasma cholesterol and alanine aminotransferase [293]. In a model of obesity and non-alcoholic fatty liver disease, EGCG significantly improved liver lipid deposition, glucose metabolism, inflammation, and liver fibrosis [294]. A similar effect on obesity and metabolic syndrome was also published in other animal studies [295,296,297]. In obese humans, EGCG supplement for 4 or 8 weeks decreased neither the body weight, nor the anthropometric measures, nor total body fat mass or percentage. However, it decreased plasma triglycerides and blood pressure [298]. In other clinical trials, EGCG supplementation for 6 weeks decreased LDL cholesterol and increased leptin but did not change any other biological parameters [299]. In contrast, EGCG treatment with high doses of EGCG led to significant weight loss, reduced waist circumference, and a consistent decline in total cholesterol and LDL plasma levels without any side effects or adverse effects in women with central obesity [300].

The benefits of EGCG in diabetes mellitus may originate from the fact that polyphenols play a significant role in carbohydrate metabolism by inhibiting key enzymes responsible for the digestion of carbohydrates to glucose such as α-glucosidase and α-amylase. EGCG enhances glucose uptake in the muscles and adipocytes by translocating GLUT4 to the plasma membrane mainly by the activation of the AMPK pathway and prevents insulin resistance [201,301]. In a murine model of type 2 diabetes, EGCG improved high-fat-diet-induced glucose tolerance and prevented NLRP3-inflammasome-dependent inflammation suggesting that EGCG as an inhibitor of NLRP3 inflammasome activation could improve glucose tolerance [302]. However, results of clinical trials are rather inconsistent. While one study demonstrated that people who drink at least four cups of tea *per* day may have a 16% lower risk of developing type 2 diabetes [303], another study showed no prospective association of moderate intake of tea (more than three cups/day) with incidence of type 2 diabetes [304].

Hyperglycemia and insulin resistance are associated with impaired activity in the PI3K/Akt pathway that results in the deregulation of signaling reactions involved in the NO production and endothelial protection [305]. The compensatory hyperinsulinemia can subsequently stimulate the MAPK pathway which may increase an ET-1 release and cause endothelial dysfunction and pro-inflammatory predisposition to pro-thrombotic and pro-atherogenic vascular events [306]. EGCG may partially prevent diabetes-associated complications by influencing the above-mentioned pathways [200] and may attenuate high glucose-induced endothelial cell inflammation via suppression of PKC and NF-kB signaling [307], as well.

## 5. Conclusions

As previously demonstrated in numerous epidemiological studies, drinking green tea has appeared to be beneficial for the prevention of various diseases, particularly cancer, and neurological, cardiovascular, respiratory, and metabolic disorders. However, the biological effects of individual green tea polyphenols including EGCG have not been completely elucidated. Thanks to multiple interactions with cell surface receptors, intracellular signaling pathways, and nuclear transcription factors, EGCG possesses a wide variety of anti-inflammatory, antioxidant, antifibrotic, anti-remodelation, and tissue-protective properties which may be useful in treatment of the above-mentioned diseases. Nevertheless, further research is necessary to find out appropriate dosing regimens and novel formulations of EGCG delivery to supply adequate local concentrations of EGCG in the tissues. In addition, the potential adverse effects of high doses of EGCG as well as possible interactions with other simultaneously delivered treatments should be evaluated before the use of EGCG may be recommended.

## References

[1] Lin Y., Jiang M., Chen W., Zhao T., Wei Y. (2019). Cancer and ER stress: Mutual crosstalk between autophagy, oxidative stress and inflammatory response. Biomed. Pharmacother..

[2] Kay J., Thadhani E., Samson L., Engelward B. (2019). Inflammation-induced DNA damage, mutations and cancer. DNA Repair.

[3] Steven S., Frenis K., Oelze M., Kalinovic S., Kuntic M., Bayo Jimenez M.T., Vujacic-Mirski K., Helmstädter J., Kröller-Schön S., Münzel T. (2019). Vascular Inflammation and Oxidative Stress: Major Triggers for Cardiovascular Disease. Oxid. Med. Cell. Longev..

[4] Marchio P., Guerra-Ojeda S., Vila J.M., Aldasoro M., Victor V.M., Mauricio M.D. (2019). Targeting Early Atherosclerosis: A Focus on Oxidative Stress and Inflammation. Oxid. Med. Cell. Longev..

[5] Luc K., Schramm-Luc A., Guzik T.J., Mikolajczyk T.P. (2019). Oxidative stress and inflammatory markers in prediabetes and diabetes. J. Physiol. Pharmacol..

[6] Halim M., Halim A. (2019). The effects of inflammation, aging and oxidative stress on the pathogenesis of diabetes mellitus (type 2 diabetes). Diabetes Metab. Syndr..

[7] Daenen K., Andries A., Mekahli D., Van Schepdael A., Jouret F., Bammens B. (2019). Oxidative stress in chronic kidney disease. Pediatr. Nephrol..

[8] Stenvinkel P., Chertow G.M., Devarajan P., Levin A., Andreoli S.P., Bangalore S., Warady B.A. (2021). Chronic Inflammation in Chronic Kidney Disease Progression: Role of Nrf2. Kidney Int. Rep..

[9] Simpson D.S.A., Oliver P.L. (2020). ROS Generation in Microglia: Understanding Oxidative Stress and Inflammation in Neurodegenerative Disease. Antioxidants.

[10] Ju Y., Tam K.Y. (2022). Pathological mechanisms and therapeutic strategies for Alzheimer’s disease. Neural Regen. Res..

[11] Mokrá D. (2020). Acute lung injury—From pathophysiology to treatment. Physiol. Res..

[12] Alam M.S., Czajkowsky D.M. (2022). SARS-CoV-2 infection and oxidative stress: Pathophysiological insight into thrombosis and therapeutic opportunities. Cytokine Growth Factor Rev..

[13] Kirkham P.A., Barnes P.J. (2013). Oxidative stress in COPD. Chest.

[14] Barnes P.J. (2016). Inflammatory mechanisms in patients with chronic obstructive pulmonary disease. J. Allergy Clin. Immunol..

[15] Barnes P.J. (2017). Cellular and molecular mechanisms of asthma and COPD. Clin. Sci..

[16] Michaeloudes C., Abubakar-Waziri H., Lakhdar R., Raby K., Dixey P., Adcock I.M., Mumby S., Bhavsar P.K., Chung K.F. (2022). Molecular mechanisms of oxidative stress in asthma. Mol. Asp. Med..

[17] Cheresh P., Kim S.J., Tulasiram S., Kamp D.W. (2013). Oxidative stress and pulmonary fibrosis. Biochim. Biophys. Acta..

[18] Phan T.H.G., Paliogiannis P., Nasrallah G.K., Giordo R., Eid A.H., Fois A.G., Zinellu A., Mangoni A.A., Pintus G. (2021). Emerging cellular and molecular determinants of idiopathic pulmonary fibrosis. Cell. Mol. Life Sci..

[19] Castro M.D.C., Pereira C.A.C. (2020). Nonlife-Threatening Sarcoidosis. Semin. Respir. Crit. Care Med..

[20] Fois S.S., Canu S., Fois A.G. (2021). The Role of Oxidative Stress in Sarcoidosis. Int. J. Mol. Sci..

[21] Adamcakova J., Mokra D. (2021). New Insights into Pathomechanisms and Treatment Possibilities for Lung Silicosis. Int. J. Mol. Sci..

[22] Tan S., Chen S. (2021). Macrophage Autophagy and Silicosis: Current Perspective and Latest Insights. Int. J. Mol. Sci..

[23] Fischer B.M., Voynow J.A., Ghio A.J. (2015). COPD: Balancing oxidants and antioxidants. Int. J. Chronic Obstr. Pulm. Dis. Int..

[24] Mishra V., Banga J., Silveyra P. (2018). Oxidative stress and cellular pathways of asthma and inflammation: Therapeutic strategies and pharmacological targets. Pharmacol. Ther..

[25] Neha K., Haider M.R., Pathak A., Yar M.S. (2019). Medicinal prospects of antioxidants: A review. Eur. J. Med. Chem..

[26] Román G.C., Jackson R.E., Gadhia R., Román A.N., Reis J. (2019). Mediterranean diet: The role of long-chain ω-3 fatty acids in fish; polyphenols in fruits, vegetables, cereals, coffee, tea, cacao and wine; probiotics and vitamins in prevention of stroke, age-related cognitive decline, and Alzheimer disease. Rev. Neurol..

[27] Dastmalchi N., Baradaran B., Latifi-Navid S., Safaralizadeh R., Khojasteh S.M.B., Amini M., Roshani E., Lotfinejad P. (2020). Antioxidants with two faces toward cancer. Life Sci..

[28] Jenkins D.J.A., Kitts D., Giovannucci E.L., Sahye-Pudaruth S., Paquette M., Blanco Mejia S., Patel D., Kavanagh M., Tsirakis T., Kendall C.W.C. (2020). Selenium, antioxidants, cardiovascular disease, and all-cause mortality: A systematic review and meta-analysis of randomized controlled trials. Am. J. Clin. Nutr..

[29] Lorenzon Dos Santos J., Quadros A.S., Weschenfelder C., Garofallo S.B., Marcadenti A. (2020). Oxidative Stress Biomarkers, Nut-Related Antioxidants, and Cardiovascular Disease. Nutrients.

[30] Soto M.E., Guarner-Lans V., Soria-Castro E., Manzano Pech L., Pérez-Torres I. (2020). Is Antioxidant Therapy a Useful Complementary Measure for Covid-19 Treatment? An Algorithm for Its Application. Medicina.

[31] Zhang P., Li T., Wu X., Nice E.C., Huang C., Zhang Y. (2020). Oxidative stress and diabetes: Antioxidative strategies. Front. Med..

[32] Gregory J., Vengalasetti Y.V., Bredesen D.E., Rao R.V. (2021). Neuroprotective Herbs for the Management of Alzheimer’s Disease. Biomolecules.

[33] Liu Y., Zhou S., Xiang D., Ju L., Shen D., Wang X., Wang Y. (2021). Friend or Foe? The Roles of Antioxidants in Acute Lung Injury. Antioxidants.

[34] Maiuolo J., Gliozzi M., Carresi C., Musolino V., Oppedisano F., Scarano F., Nucera S., Scicchitano M., Bosco F., Macri R. (2021). Nutraceuticals and Cancer: Potential for Natural Polyphenols. Nutrients.

[35] Adamcakova J., Mokra D. (2021). Herbal compounds in the treatment of pulmonary silicosis. Physiol. Res..

[36] Muchtaridi M., Amirah S.R., Harmonis J.A., Ikram E.H.K. (2022). Role of Nuclear Factor Erythroid 2 (Nrf2) in the Recovery of Long COVID-19 Using Natural Antioxidants: A Systematic Review. Antioxidants.

[37] von Knethen A., Heinicke U., Laux V., Parnham M.J., Steinbicker A.U., Zacharowski K. (2022). Antioxidants as Therapeutic Agents in Acute Respiratory Distress Syndrome (ARDS) Treatment-From Mice to Men. Biomedicines.

[38] Lago J.H., Toledo-Arruda A.C., Mernak M., Barrosa K.H., Martins M.A., Tibério I.F., Prado C.M. (2014). Structure-activity association of flavonoids in lung diseases. Molecules.

[39] Singh B.N., Shankar S., Srivastava R.K. (2011). Green tea catechin, epigallocatechin-3-gallate (EGCG): Mechanisms, perspectives and clinical applications. Biochem. Pharmacol..

[40] Kim H.S., Quon M.J., Kim J.A. (2014). New insights into the mechanisms of polyphenols beyond antioxidant properties; lessons from the green tea polyphenol, epigallocatechin 3-gallate. Redox Biol..

[41] Todoric J., Antonucci L., Karin M. (2016). Targeting Inflammation in Cancer Prevention and Therapy. Cancer Prev. Res..

[42] Almatroodi S.A., Almatroudi A., Khan A.A., Alhumaydhi F.A., Alsahli M.A., Rahmani A.H. (2020). Potential Therapeutic Targets of Epigallocatechin Gallate (EGCG), the Most Abundant Catechin in Green Tea, and Its Role in the Therapy of Various Types of Cancer. Molecules.

[43] Yamagata K. (2020). Protective Effect of Epigallocatechin Gallate on Endothelial Disorders in Atherosclerosis. J. Cardiovasc. Pharmacol..

[44] Mukhtar H., Ahmad N. (2000). Tea polyphenols: Prevention of cancer and optimizing health. Am. J. Clin. Nutr..

[45] Balentine D.A., Wiseman S.A., Bouwens L.C. (1997). The chemistry of tea flavonoids. Crit. Rev. Food Sci. Nutr..

[46] Khan N., Afaq F., Saleem M., Ahmad N., Mukhtar H. (2006). Targeting multiple signaling pathways by green tea polyphenol (-)-epigallocatechin-3-gallate. Cancer Res..

[47] https://en.wikipedia.org/wiki/Camellia_sinensis.

[48] https://en.wikipedia.org/wiki/Green_tea.

[49] https://en.wikipedia.org/wiki/Epigallocatechin_gallate.

[50] Henning S.M., Niu Y., Lee N.H., Thames G.D., Minutti R.R., Wang H., Go V.L., Heber D. (2004). Bioavailability and antioxidant activity of tea flavanols after consumption of green tea, black tea, or a green tea extract supplement. Am. J. Clin. Nutr..

[51] https://en.wikipedia.org/wiki/Epicatechin_gallate.

[52] Zwolak I. (2021). Epigallocatechin Gallate for Management of Heavy Metal-Induced Oxidative Stress: Mechanisms of Action, Efficacy, and Concerns. Int. J. Mol. Sci..

[53] Van Amelsvoort J.M., Van Hof K.H., Mathot J.N., Mulder T.P., Wiersma A., Tijburg L.B. (2001). Plasma concentrations of individual tea catechins after a single oral dose in humans. Xenobiotica.

[54] Nanjo F., Mori M., Goto K., Hara Y. (1999). Radical scavenging activity of tea catechins and their related compounds. Biosci. Biotechnol. Biochem..

[55] Higdon J.V., Frei B. (2003). Tea catechins and polyphenols: Health effects, metabolism, and antioxidant functions. Crit. Rev. Food Sci. Nutr..

[56] Lambert J.D., Lee M.J., Diamond L., Ju J., Hong J., Bose M., Newmark H.L., Yang C.S. (2006). Dose-dependent levels of epigallocatechin-3-gallate in human colon cancer cells and mouse plasma and tissues. Drug Metab. Dispos..

[57] Collins Q.F., Liu H.Y., Pi J., Liu Z., Quon M.J., Cao W. (2007). Epigallocatechin-3-gallate (EGCG), a green tea polyphenol, suppresses hepatic gluconeogenesis through 5’-AMP-activated protein kinase. J. Biol. Chem..

[58] Elbling L., Herbacek I., Weiss R.M., Jantschitsch C., Micksche M., Gerner C., Pangratz H., Grusch M., Knasmüller S., Berger W. (2010). Hydrogen peroxide mediates EGCG-induced antioxidant protection in human keratinocytes. Free Radic. Biol. Med..

[59] Nakagawa H., Wachi M., Woo J.T., Kato M., Kasai S., Takahashi F., Lee I.S., Nagai K. (2002). Fenton reaction is primarily involved in a mechanism of (-)-epigallocatechin-3-gallate to induce osteoclastic cell death. Biochem. Biophys. Res. Commun..

[60] Nakagawa H., Hasumi K., Woo J.T., Nagai K., Wachi M. (2004). Generation of hydrogen peroxide primarily contributes to the induction of Fe(II)-dependent apoptosis in Jurkat cells by (-)-epigallocatechin gallate. Carcinogenesis.

[61] Satoh M., Takemura Y., Hamada H., Sekido Y., Kubota S. (2013). EGCG induces human mesothelioma cell death by inducing reactive oxygen species and autophagy. Cancer Cell Int..

[62] Krupkova O., Ferguson S.J., Wuertz-Kozak K. (2016). Stability of (-)-epigallocatechin gallate and its activity in liquid formulations and delivery systems. J. Nutr. Biochem..

[63] Rietveld A., Wiseman S. (2003). Antioxidant effects of tea: Evidence from human clinical trials. J. Nutr..

[64] Toniolo A., Buccellati C., Pinna C., Gaion R.M., Sala A., Bolego C. (2013). Cyclooxygenase-1 and prostacyclin production by endothelial cells in the presence of mild oxidative stress. PLoS ONE.

[65] Lambert J.D., Sang S., Yang C.S. (2007). Biotransformation of green tea polyphenols and the biological activities of those metabolites. Mol. Pharm..

[66] Timmel M.A., Byl J.A., Osheroff N. (2013). Epimerization of green tea catechins during brewing does not affect the ability to poi-son human type II topoisomerases. Chem. Res. Toxicol..

[67] Negri A., Naponelli V., Rizzi F., Bettuzzi S. (2018). Molecular Targets of Epigallocatechin-Gallate (EGCG): A Special Focus on Signal Transduction and Cancer. Nutrients.

[68] Tachibana H., Koga K., Fujimura Y., Yamada K. (2004). A receptor for green tea polyphenol EGCG. Nat. Struct. Mol. Biol..

[69] Umeda D., Yano S., Yamada K., Tachibana H. (2008). Green tea polyphenol epigallocatechin-3-gallate signaling pathway through 67-kDa laminin receptor. J. Biol. Chem..

[70] Kumazoe M., Sugihara K., Tsukamoto S., Huang Y., Tsurudome Y., Suzuki T., Suemasu Y., Ueda N., Yamashita S., Kim Y. (2013). 67-kDa laminin receptor increases cGMP to induce cancer-selective apoptosis. J. Clin. Investig..

[71] Fujimura Y., Kumazoe M., Tachibana H. (2022). 67-kDa Laminin Receptor-Mediated Cellular Sensing System of Green Tea Polyphenol EGCG and Functional Food Pairing. Molecules.

[72] Yang C.S., Wang H. (2013). Cancer therapy combination: Green tea and a phosphodiesterase 5 inhibitor?. J. Clin. Investig..

[73] Hong Byun E., Fujimura Y., Yamada K., Tachibana H. (2010). TLR4 signaling inhibitory pathway induced by green tea polyphenol epigallocatechin-3-gallate through 67-kDa laminin receptor. J. Immunol..

[74] Xu M.J., Liu B.J., Wang C.L., Wang G.H., Tian Y., Wang S.H., Li J., Li P.Y., Zhang R.H., Wei D. (2017). Epigallocatechin-3-gallate inhibits TLR4 signaling through the 67-kDa laminin receptor and effectively alleviates acute lung injury induced by H9N2 swine influenza virus. Int. Immunopharmacol..

[75] O’Neill L.A., Golenbock D., Bowie A.G. (2013). The history of Toll-like receptors—Redefining innate immunity. Nat. Rev. Immunol..

[76] Byun E.B., Yang M.S., Kim J.H., Song D.S., Lee B.S., Park J.N., Park S.H., Park C., Jung P.M., Sung N.Y. (2014). Epigallocatechin-3-gallate-mediated Tollip induction through the 67-kDa laminin receptor negatively regulating TLR4 signaling in endothelial cells. Immunobiology.

[77] Chen C.Y., Kao C.L., Liu C.M. (2018). The Cancer Prevention, Anti-Inflammatory and Anti-Oxidation of Bioactive Phytochemicals Targeting the TLR4 Signaling Pathway. Int. J. Mol. Sci..

[78] Zhong X., Liu M., Yao W., Du K., He M., Jin X., Jiao L., Ma G., Wei B., Wei M. (2019). Epigallocatechin-3-Gallate Attenuates Microglial Inflammation and Neurotoxicity by Suppressing the Activation of Canonical and Noncanonical Inflammasome via TLR4/NF-κB Pathway. Mol. Nutr. Food Res..

[79] Baek C.H., Kim H., Moon S.Y., Park S.K., Yang W.S. (2019). Epigallocatechin-3-gallate downregulates lipopolysaccharide signaling in human aortic endothelial cells by inducing ectodomain shedding of TLR4. Eur. J. Pharmacol..

[80] Wang J., Fan S.M., Zhang J. (2019). Epigallocatechin-3-gallate ameliorates lipopolysaccharide-induced acute lung injury by suppression of TLR4/NF-κB signaling activation. Braz. J. Med. Biol. Res..

[81] Bao S., Cao Y., Fan C., Fan Y., Bai S., Teng W., Shan Z. (2014). Epigallocatechin gallate improves insulin signaling by decreasing toll-like receptor 4 (TLR4) activity in adipose tissues of high-fat diet rats. Mol. Nutr. Food Res..

[82] Hou H., Yang W., Bao S., Cao Y. (2020). Epigallocatechin Gallate Suppresses Inflammatory Responses by Inhibiting Toll-like Receptor 4 Signaling and Alleviates Insulin Resistance in the Livers of High-fat-diet Rats. J. Oleo Sci..

[83] Lee M.H., Kwon B.J., Koo M.A., You K.E., Park J.C. (2013). Mitogenesis of vascular smooth muscle cell stimulated by platelet-derived growth factor-bb is inhibited by blocking of intracellular signaling by epigallocatechin-3-O-gallate. Oxidative Med. Cell. Longev..

[84] Minnelli C., Cianfruglia L., Laudadio E., Mobbili G., Galeazzi R., Armeni T. (2021). Effect of Epigallocatechin-3-Gallate on EGFR Signaling and Migration in Non-Small Cell Lung Cancer. Int. J. Mol. Sci..

[85] Adachi S., Shimizu M., Shirakami Y., Yamauchi J., Natsume H., Matsushima-Nishiwaki R., To S., Weinstein I.B., Moriwaki H., Kozawa O. (2009). (-)-Epigallocatechin gallate downregulates EGF receptor via phosphorylation at Ser1046/1047 by p38 MAPK in colon cancer cells. Carcinogenesis.

[86] Zhu W., Li M.C., Wang F.R., Mackenzie G.G., Oteiza P.I. (2020). The inhibitory effect of ECG and EGCG dimeric procyanidins on colorectal cancer cells growth is associated with their actions at lipid rafts and the inhibition of the epidermal growth factor receptor signaling. Biochem. Pharmacol..

[87] Farabegoli F., Govoni M., Spisni E., Papi A. (2017). EGFR inhibition by (-)-epigallocatechin-3-gallate and IIF treatments reduces breast cancer cell invasion. Biosci. Rep..

[88] Rashidi B., Malekzadeh M., Goodarzi M., Masoudifar A., Mirzaei H. (2017). Green tea and its anti-angiogenesis effects. Biomed. Pharmacother..

[89] Shimizu M., Shirakami Y., Sakai H., Yasuda Y., Kubota M., Adachi S., Tsurumi H., Hara Y., Moriwaki H. (2010). (-)-Epigallocatechin gallate inhibits growth and activation of the VEGF/VEGFR axis in human colorectal cancer cells. Chem. Biol. Interact..

[90] Shirakami Y., Shimizu M., Adachi S., Sakai H., Nakagawa T., Yasuda Y., Tsurumi H., Hara Y., Moriwaki H. (2009). (-)-Epigallocatechin gallate suppresses the growth of human hepatocellular carcinoma cells by inhibiting activation of the vascular endothelial growth factor-vascular endothelial growth factor receptor axis. Cancer Sci..

[91] Shimizu M., Deguchi A., Lim J.T., Moriwaki H., Kopelovich L., Weinstein I.B. (2005). (−) Epigallocatechin gallate and polyphenon E inhibit growth and activation of the epidermal growth factor receptor and human epidermal growth factor receptor-2 signaling pathways in human colon cancer cells. Clin. Cancer Res..

[92] Shimizu M., Shirakami Y., Sakai H., Tatebe H., Nakagawa T., Hara Y., Weinstein I.B., Moriwaki H. (2008). EGCG inhibits activation of the insulin-like growth factor (IGF)/IGF-1 receptor axis in human hepatocellular carcinoma cells. Cancer Lett..

[93] Vu H.A., Beppu Y., Chi H.T., Sasaki K., Yamamoto H., Xinh P.T., Tanii T., Hara Y., Watanabe T., Sato Y. (2010). Green tea epigallocatechin gallate exhibits anticancer effect in human pancreatic carcinoma cells via the inhibition of both focal adhesion kinase and insulin-like growth factor-I receptor. J. Biomed. Biotechnol..

[94] Della Via F.I., Shiraishi R.N., Santos I., Ferro K.P., Salazar-Terreros M.J., Franchi Junior G.C., Rego E.M., Saad S.T.O., Torello C.O. (2021). (-)-Epigallocatechin-3-gallate induces apoptosis and differentiation in leukaemia by targeting reactive oxygen species and PIN1. Sci. Rep..

[95] Alvarez E., Campos-Toimil M., Justiniano-Basaran H., Lugnier C., Orallo F. (2006). Study of the mechanisms involved in the vasorelaxation induced by (-)-epigallocatechin-3-gallate in rat aorta. Br. J. Pharmacol..

[96] Hotta Y., Huang L., Muto T., Yajima M., Miyazeki K., Ishikawa N., Fukuzawa Y., Wakida Y., Tushima H., Ando H. (2006). Positive inotropic effect of purified green tea catechin derivative in guinea pig hearts: The measurements of cellular Ca2+ and nitric oxide release. Eur. J. Pharmacol..

[97] Ok W.J., Cho H.J., Kim H.H., Lee D.H., Kang H.Y., Kwon H.W., Rhee M.H., Kim M., Park H.J. (2012). Epigallocatechin-3-gallate has an anti-platelet effect in a cyclic AMP-dependent manner. J. Atheroscler. Thromb..

[98] Liang Y., Ip M.S.M., Mak J.C.W. (2019). (-)-Epigallocatechin-3-gallate suppresses cigarette smoke-induced inflammation in human cardiomyocytes via ROS-mediated MAPK and NF-κB pathways. Phytomedicine.

[99] Liu C., Hao K., Liu Z., Liu Z., Guo N. (2021). Epigallocatechin gallate (EGCG) attenuates staphylococcal alpha-hemolysin (Hla)-induced NLRP3 inflammasome activation via ROS-MAPK pathways and EGCG-Hla interactions. Int. Immunopharmacol..

[100] Cerezo-Guisado M.I., Zur R., Lorenzo M.J., Risco A., Martín-Serrano M.A., Alvarez-Barrientos A., Cuenda A., Centeno F. (2015). Implication of Akt, ERK1/2 and alternative p38MAPK signalling pathways in human colon cancer cell apoptosis induced by green tea EGCG. Food Chem. Toxicol..

[101] Zhou Y., Tang J., Du Y., Ding J., Liu J.Y. (2016). The green tea polyphenol EGCG potentiates the antiproliferative activity of sunitinib in human cancer cells. Tumour Biol..

[102] He Y., Yang Z., Pi J., Cai T., Xia Y., Cao X., Liu J. (2022). EGCG attenuates the neurotoxicity of methylglyoxal via regulating MAPK and the downstream signaling pathways and inhibiting advanced glycation end products formation. Food Chem..

[103] Shimizu M., Deguchi A., Joe A.K., Mckoy J.F., Moriwaki H., Weinstein I.B. (2005). EGCG inhibits activation of HER3 and expression of cyclooxygenase-2 in human colon cancer cells. J. Exp. Ther. Oncol..

[104] Hussain T., Gupta S., Adhami V.M., Mukhtar H. (2005). Green tea constituent epigallocatechin-3-gallate selectively inhibits COX-2 without affecting COX-1 expression in human prostate carcinoma cells. Int. J. Cancer.

[105] Zhong Y., Chiou Y.S., Pan M.H., Shahidi F. (2012). Anti-inflammatory activity of lipophilic epigallocatechin gallate (EGCG) derivatives in LPS-stimulated murine macrophages. Food Chem..

[106] Lai S.W., Chen J.H., Lin H.Y., Liu Y.S., Tsai C.F., Chang P.C., Lu D.Y., Lin C. (2018). Regulatory Effects of Neuroinflammatory Responses Through Brain-Derived Neurotrophic Factor Signaling in Microglial Cells. Mol. Neurobiol..

[107] Hwang J.T., Ha J., Park I.J., Lee S.K., Baik H.W., Kim Y.M., Park O.J. (2007). Apoptotic effect of EGCG in HT-29 colon cancer cells via AMPK signal pathway. Cancer Lett..

[108] Huang C.H., Tsai S.J., Wang Y.J., Pan M.H., Kao J.Y., Way T.D. (2009). EGCG inhibits protein synthesis, lipogenesis, and cell cycle progression through activation of AMPK in p53 positive and negative human hepatoma cells. Mol. Nutr. Food Res..

[109] Zhou J., Farah B.L., Sinha R.A., Wu Y., Singh B.K., Bay B.H., Yang C.S., Yen P.M. (2014). Epigallocatechin-3-gallate (EGCG), a green tea polyphenol, stimulates hepatic autophagy and lipid clearance. PLoS ONE.

[110] Peairs A., Dai R., Gan L., Shimp S., Rylander M.N., Li L., Reilly C.M. (2010). Epigallocatechin-3-gallate (EGCG) attenuates inflammation in MRL/lpr mouse mesangial cells. Cell. Mol. Immunol..

[111] Wu Y., Cui J. (2020). (-)-Epigallocatechin-3-gallate provides neuroprotection via AMPK activation against traumatic brain injury in a mouse model. Naunyn. Schmiedebergs. Arch. Pharmacol..

[112] Kim J.E., Park H., Jeong M.J., Kang T.C. (2020). Epigallocatechin-3-Gallate and PEDF 335 Peptide, 67LR Activators, Attenuate Vasogenic Edema, and Astroglial Degeneration Following Status Epilepticus. Antioxidants.

[113] Yang N., Zhang H., Cai X., Shang Y. (2018). Epigallocatechin-3-gallate inhibits inflammation and epithelial-mesenchymal transition through the PI3K/AKT pathway via upregulation of PTEN in asthma. Int. J. Mol. Med..

[114] Nan W., Zhonghang X., Keyan C., Tongtong L., Wanshu G., Zhongxin X. (2018). Epigallocatechin-3-Gallate Reduces Neuronal Apoptosis in Rats after Middle Cerebral Artery Occlusion Injury via PI3K/AKT/eNOS Signaling Pathway. Biomed. Res. Int..

[115] Zhang Z., Zhang D. (2020). (-)-Epigallocatechin-3-Gallate Inhibits eNOS Uncoupling and Alleviates High Glucose-Induced Dysfunction and Apoptosis of Human Umbilical Vein Endothelial Cells by PI3K/AKT/eNOS Pathway. Diabetes Metab. Syndr. Obes..

[116] Reddy A.T., Lakshmi S.P., Maruthi Prasad E., Varadacharyulu N.C., Kodidhela L.D. (2020). Epigallocatechin gallate suppresses inflammation in human coronary artery endothelial cells by inhibiting NF-κB. Life Sci..

[117] Lakshmi S.P., Reddy A.T., Kodidhela L.D., Varadacharyulu N.C. (2020). Epigallocatechin gallate diminishes cigarette smoke-induced oxidative stress, lipid peroxidation, and inflammation in human bronchial epithelial cells. Life Sci..

[118] Luo K.W., Chen W., Lung W.Y., Wei X.Y., Cheng B.H., Cai Z.M., Huang W.R. (2017). EGCG inhibited bladder cancer SW780 cell proliferation and migration both in vitro and in vivo via down-regulation of NF-κB and MMP-9. J. Nutr. Biochem..

[119] Kim S.R., Seong K.J., Kim W.J., Jung J.Y. (2022). Epigallocatechin Gallate Protects against Hypoxia-Induced Inflammation in Microglia via NF-κB Suppression and Nrf-2/HO-1 Activation. Int. J. Mol. Sci..

[120] Huang S.C., Kao Y.H., Shih S.F., Tsai M.C., Lin C.S., Chen L.W., Chuang Y.P., Tsui P.F., Ho L.J., Lai J.H. (2021). Epigallocatechin-3-gallate exhibits immunomodulatory effects in human primary T cells. Biochem. Biophys. Res. Commun..

[121] Aneja R., Hake P.W., Burroughs T.J., Denenberg A.G., Wong H.R., Zingarelli B. (2004). Epigallocatechin, a green tea polyphenol, attenuates myocardial ischemia reperfusion injury in rats. Mol. Med..

[122] Nair S., Barve A., Khor T.O., Shen G.X., Lin W., Chan J.Y., Cai L., Kong A.N. (2010). Regulation of Nrf2- and AP-1-mediated gene expression by epigallocatechin-3-gallate and sulforaphane in prostate of Nrf2-knockout or C57BL/6J mice and PC-3 AP-1 human prostate cancer cells. Acta Pharmacol. Sin..

[123] You H., Wei L., Sun W.L., Wang L., Yang Z.L., Liu Y., Zheng K., Wang Y., Zhang W.J. (2014). The green tea extract epigallocatechin-3-gallate inhibits irradiation-induced pulmonary fibrosis in adult rats. Int. J. Mol. Med..

[124] Zhang Q., Liu J., Duan H., Li R., Peng W., Wu C. (2021). Activation of Nrf2/HO-1 signaling: An important molecular mechanism of herbal medicine in the treatment of atherosclerosis via the protection of vascular endothelial cells from oxidative stress. J. Adv. Res..

[125] Huang Y.J., Wang K.L., Chen H.Y., Chiang Y.F., Hsia S.M. (2020). Protective Effects of Epigallocatechin Gallate (EGCG) on Endometrial, Breast, and Ovarian Cancers. Biomolecules.

[126] Datta S., Ghosh S., Bishayee A., Sinha D. (2022). Flexion of Nrf2 by tea phytochemicals: A review on the chemopreventive and chemotherapeutic implications. Pharmacol. Res..

[127] Smith R.E., Tran K., Smith C.C., McDonald M., Shejwalkar P., Hara K. (2016). The Role of the Nrf2/ARE Antioxidant System in Preventing Cardiovascular Diseases. Diseases.

[128] Leonardo C.C., Doré S. (2011). Dietary flavonoids are neuroprotective through Nrf2-coordinated induction of endogenous cytoprotective proteins. Nutr. Neurosci..

[129] Townsend P.A., Scarabelli T.M., Pasini E., Gitti G., Menegazzi M., Suzuki H., Knight R.A., Latchman D.S., Stephanou A. (2004). Epigallocatechin-3-gallate inhibits STAT-1 activation and protects cardiac myocytes from ischemia/reperfusion-induced apoptosis. FASEB J..

[130] Ogawa K., Hara T., Shimizu M., Nagano J., Ohno T., Hoshi M., Ito H., Tsurumi H., Saito K., Seishima M. (2012). (-)-Epigallocatechin gallate inhibits the expression of indoleamine 2,3-dioxygenase in human colorectal cancer cells. Oncol. Lett..

[131] Senggunprai L., Kukongviriyapan V., Prawan A., Kukongviriyapan U. (2014). Quercetin and EGCG exhibit chemopreventive effects in cholangiocarcinoma cells via suppression of JAK/STAT signaling pathway. Phytother. Res..

[132] Menegazzi M., Tedeschi E., Dussin D., De Prati A.C., Cavalieri E., Mariotto S., Suzuki H. (2001). Anti-interferon gamma action of epigallocatechin-3-gallate mediated by specific inhibition of STAT1 activation. FASEB J..

[133] Liu W., Dong M., Bo L., Li C., Liu Q., Li Y., Ma L., Xie Y., Fu E., Mu D. (2014). Epigallocatechin-3-gallate ameliorates seawater aspiration-induced acute lung injury via regulating inflammatory cytokines and inhibiting JAK/STAT1 pathway in rats. Mediat. Inflamm..

[134] Tang Y., Matsuoka I., Ono T., Inoue K., Kimura J. (2008). Selective up-regulation of P2X4-receptor gene expression by interferon-gamma in vascular endothelial cells. J. Pharmacol. Sci..

[135] Lee I.T., Lin C.C., Lee C.Y., Hsieh P.W., Yang C.M. (2013). Protective effects of (-)-epigallocatechin-3-gallate against TNF-α-induced lung inflammation via ROS-dependent ICAM-1 inhibition. J. Nutr. Biochem..

[136] Kryczek I., Lin Y., Nagarsheth N., Peng D., Zhao L., Zhao E., Vatan L., Szeliga W., Dou Y., Owens S. (2014). IL-22(+)CD4(+) T cells promote colorectal cancer stemness via STAT3 transcription factor activation and induction of the methyltransferase DOT1L. Immunity.

[137] Yang G.Y., Liao J., Li C., Chung J., Yurkow E.J., Ho C.T., Yang C.S. (2000). Effect of black and green tea polyphenols on c-jun phosphorylation and H(2)O(2) production in transformed and non-transformed human bronchial cell lines: Possible mechanisms of cell growth inhibition and apoptosis induction. Carcinogenesis.

[138] Li G.X., Chen Y.K., Hou Z., Xiao H., Jin H., Lu G., Lee M.J., Liu B., Guan F., Yang Z. (2010). Pro-oxidative activities and dose-response relationship of (-)-epigallocatechin-3-gallate in the inhibition of lung cancer cell growth: A comparative study in vivo and in vitro. Carcinogenesis.

[139] Lorenz M., Wessler S., Follmann E., Michaelis W., Düsterhöft T., Baumann G., Stangl K., Stangl V. (2004). A constituent of green tea, epigallocatechin-3-gallate, activates endothelial nitric oxide synthase by a phosphatidylinositol-3-OH-kinase-, cAMP-dependent protein kinase-, and Akt-dependent pathway and leads to endothelial-dependent vasorelaxation. J. Biol. Chem..

[140] Pearson G., Robinson F., Beers Gibson T., Xu B.E., Karandikar M., Berman K., Cobb M.H. (2001). Mitogen-activated protein (MAP) kinase pathways: Regulation and physiological functions. Endocr. Rev..

[141] https://en.wikipedia.org/wiki/Mitogen-activated_protein_kinase#/media/File:MAPK-pathway-mammalian.png.

[142] Xu D., Peng S., Guo R., Yao L., Mo H., Li H., Song H., Hu L. (2021). EGCG Alleviates Oxidative Stress and Inhibits Aflatoxin B1 Biosynthesis via MAPK Signaling Pathway. Toxins.

[143] Mou Q., Jiang Y., Zhu L., Zhu Z., Ren T. (2020). EGCG induces β-defensin 3 against influenza A virus H1N1 by the MAPK signaling pathway. Exp. Ther. Med..

[144] Kim H.S., Kim M.H., Jeong M., Hwang Y.S., Lim S.H., Shin B.A., Ahn B.W., Jung Y.D. (2004). EGCG blocks tumor promoter-induced MMP-9 expression via suppression of MAPK and AP-1 activation in human gastric AGS cells. Anticancer Res..

[145] Chen J., Chen L., Lu T., Xie Y., Li C., Jia Z., Cao J. (2019). ERα36 is an effective target of epigallocatechin-3-gallate in hepatocellular carcinoma. Int. J. Clin. Exp. Pathol..

[146] O’Banion M.K. (1999). Cyclooxygenase-2: Molecular biology, pharmacology, and neurobiology. Crit. Rev. Neurobiol..

[147] Park I.J., Lee Y.K., Hwang J.T., Kwon D.Y., Ha J., Park O.J. (2009). Green tea catechin controls apoptosis in colon cancer cells by attenuation of H_2_O_2_-stimulated COX-2 expression via the AMPK signaling pathway at low-dose H_2_O_2_. Ann. N. Y. Acad. Sci..

[148] Singh T., Katiyar S.K. (2011). Green tea catechins reduce invasive potential of human melanoma cells by targeting COX-2, PGE2 receptors and epithelial-to-mesenchymal transition. PLoS ONE.

[149] Ye F., Zhang G.H., Guan B.X., Xu X.C. (2012). Suppression of esophageal cancer cell growth using curcumin, (-)-epigallocatechin-3-gallate and lovastatin. World J. Gastroenterol..

[150] Yang X.W., Wang X.L., Cao L.Q., Jiang X.F., Peng H.P., Lin S.M., Xue P., Chen D. (2012). Green tea polyphenol epigallocatechin-3-gallate enhances 5-fluorouracil-induced cell growth inhibition of hepatocellular carcinoma cells. Hepatol. Res..

[151] Ahmed S., Rahman A., Hasnain A., Lalonde M., Goldberg V.M., Haqqi T.M. (2002). Green tea polyphenol epigallocatechin-3-gallate inhibits the IL-1 beta-induced activity and expression of cyclooxygenase-2 and nitric oxide synthase-2 in human chondrocytes. Free Radic. Biol. Med..

[152] Jeon S.M. (2016). Regulation and function of AMPK in physiology and diseases. Exp. Mol. Med..

[153] Nicholson K.M., Anderson N.G. (2002). The protein kinase B/Akt signalling pathway in human malignancy. Cell. Signal..

[154] Gilmore T.D. (2006). Introduction to NF-kappaB: Players, pathways, perspectives. Oncogene.

[155] Arafa M.H., Atteia H.H. (2020). Protective Role of Epigallocatechin Gallate in a Rat Model of Cisplatin-Induced Cerebral Inflammation and Oxidative Damage: Impact of Modulating NF-κB and Nrf2. Neurotox. Res..

[156] Liu D., Perkins J.T., Hennig B. (2016). EGCG prevents PCB-126-induced endothelial cell inflammation via epigenetic modifications of NF-κB target genes in human endothelial cells. J. Nutr. Biochem..

[157] Syed D.N., Afaq F., Kweon M.H., Hadi N., Bhatia N., Spiegelman V.S., Mukhtar H. (2007). Green tea polyphenol EGCG suppresses cigarette smoke condensate-induced NF-kappaB activation in normal human bronchial epithelial cells. Oncogene.

[158] Surh Y.J., Chun K.S., Cha H.H., Han S.S., Keum Y.S., Park K.K., Lee S.S. (2001). Molecular mechanisms underlying chemopreventive activities of anti-inflammatory phytochemicals: Down-regulation of COX-2 and iNOS through suppression of NF-kappa B activation. Mutat. Res..

[159] Zhang L., Chen W., Tu G., Chen X., Lu Y., Wu L., Zheng D. (2020). Enhanced Chemotherapeutic Efficacy of PLGA-Encapsulated Epigallocatechin Gallate (EGCG) Against Human Lung Cancer. Int. J. Nanomed..

[160] Aggarwal B.B., Shishodia S. (2006). Molecular targets of dietary agents for prevention and therapy of cancer. Biochem. Pharmacol..

[161] Dong Z., Ma W., Huang C., Yang C.S. (1997). Inhibition of tumor promoter-induced activator protein 1 activation and cell transformation by tea polyphenols, (-)-epigallocatechin gallate, and theaflavins. Cancer Res..

[162] Sen T., Dutta A., Chatterjee A. (2010). Epigallocatechin-3-gallate (EGCG) downregulates gelatinase-B (MMP-9) by involvement of FAK/ERK/NFkappaB and AP-1 in the human breast cancer cell line MDA-MB-231. Anticancer Drugs.

[163] Kim J.E., Shin M.H., Chung J.H. (2013). Epigallocatechin-3-gallate prevents heat shock-induced MMP-1 expression by inhibiting AP-1 activity in human dermal fibroblasts. Arch. Dermatol. Res..

[164] Khoi P.N., Park J.S., Kim J.H., Xia Y., Kim N.H., Kim K.K., Jung Y.D. (2013). (-)-Epigallocatechin-3-gallate blocks nicotine-induced matrix metalloproteinase-9 expression and invasiveness via suppression of NF-κB and AP-1 in endothelial cells. Int. J. Oncol..

[165] Abboud P.A., Hake P.W., Burroughs T.J., Odoms K., O’Connor M., Mangeshkar P., Wong H.R., Zingarelli B. (2008). Therapeutic effect of epigallocatechin-3-gallate in a mouse model of colitis. Eur. J. Pharmacol..

[166] Talebi M., Talebi M., Farkhondeh T., Mishra G., İlgün S., Samarghandian S. (2021). New insights into the role of the Nrf2 signaling pathway in green tea catechin applications. Phytotherapy Res..

[167] Canning P., Sorrell F.J., Bullock A.N. (2015). Structural basis of Keap1 interactions with Nrf2. Free Radic. Biol. Med..

[168] He F., Antonucci L., Karin M. (2020). NRF2 as a regulator of cell metabolism and inflammation in cancer. Carcinogenesis.

[169] Wu K.C., Cui J.Y., Klaassen C.D. (2012). Effect of graded Nrf2 activation on phase-I and -II drug metabolizing enzymes and transporters in mouse liver. PLoS ONE.

[170] Taguchi K., Yamamoto M. (2017). The KEAP1-NRF2 System in Cancer. Front. Oncol..

[171] Kobayashi E., Suzuki T., Yamamoto M. (2013). Roles nrf2 plays in myeloid cells and related disorders. Oxid. Med. Cell. Longev..

[172] Pullikotil P., Chen H., Muniyappa R., Greenberg C.C., Yang S., Reiter C.E., Lee J.W., Chung J.H., Quon M.J. (2012). Epigallocatechin gallate induces expression of heme oxygenase-1 in endothelial cells via p38 MAPK and Nrf-2 that suppresses proinflammatory actions of TNF-α. J. Nutr. Biochem..

[173] Smith R.E. (2020). The Effects of Dietary Supplements that Overactivate the Nrf2/ARE System. Curr. Med. Chem..

[174] Baranwal A., Aggarwal P., Rai A., Kumar N. (2022). Pharmacological Actions and Underlying Mechanisms of Catechin: A Review. Mini Rev. Med. Chem..

[175] Han J., Wang M., Jing X., Shi H., Ren M., Lou H. (2014). (-)-Epigallocatechin gallate protects against cerebral ischemia-induced oxidative stress via Nrf2/ARE signaling. Neurochem. Res..

[176] Michaličková D., Hrnčíř T., Canová N.K., Slanař O. (2020). Targeting Keap1/Nrf2/ARE signaling pathway in multiple sclerosis. Eur. J. Pharmacol..

[177] Wang J., Wu Q., Ding L., Song S., Li Y., Shi L., Wang T., Zhao D., Wang Z., Li X. (2021). Therapeutic Effects and Molecular Mechanisms of Bioactive Compounds Against Respiratory Diseases: Traditional Chinese Medicine Theory and High-Frequency Use. Front. Pharmacol..

[178] Zhang Z., Zhang X., Bi K., He Y., Yan W., Yang C.S., Zhang J. (2021). Potential protective mechanisms of green tea polyphenol EGCG against COVID-19. Trends Food Sci. Technol..

[179] Suzuki T., Pervin M., Goto S., Isemura M., Nakamura Y. (2016). Beneficial Effects of Tea and the Green Tea Catechin Epigallocatechin-3-gallate on Obesity. Molecules.

[180] Sun W., Liu X., Zhang H., Song Y., Li T., Liu X., Liu Y., Guo L., Wang F., Yang T. (2017). Epigallocatechin gallate upregulates NRF2 to prevent diabetic nephropathy via disabling KEAP1. Free Radic. Biol. Med..

[181] Tang G., Xu Y., Zhang C., Wang N., Li H., Feng Y. (2021). Green Tea and Epigallocatechin Gallate (EGCG) for the Management of Nonalcoholic Fatty Liver Diseases (NAFLD): Insights into the Role of Oxidative Stress and Antioxidant Mechanism. Antioxidants.

[182] O’Shea J.J., Schwartz D.M., Villarino A.V., Gadina M., McInnes I.B., Laurence A. (2015). The JAK-STAT pathway: Impact on human disease and therapeutic intervention. Annu. Rev. Med..

[183] Xin P., Xu X., Deng C., Liu S., Wang Y., Zhou X., Ma H., Wei D., Sun S. (2020). The role of JAK/STAT signaling pathway and its inhibitors in diseases. Int. Immunopharmacol..

[184] de Prati A.C., Ciampa A.R., Cavalieri E., Zaffini R., Darra E., Menegazzi M., Suzuki H., Mariotto S. (2005). STAT1 as a new molecular target of anti-inflammatory treatment. Curr. Med. Chem..

[185] Yamamoto K., Korenaga R., Kamiya A., Ando J. (2000). Fluid shear stress activates Ca(2+) influx into human endothelial cells via P2X4 purinoceptors. Circ. Res..

[186] Ren J.L., Yu Q.X., Liang W.C., Leung P.Y., Ng T.K., Chu W.K., Pang C.P., Chan S.O. (2018). Green tea extract attenuates LPS-induced retinal inflammation in rats. Sci. Rep..

[187] Wang J., Li P., Qin T., Sun D., Zhao X., Zhang B. (2020). Protective effect of epigallocatechin-3-gallate against neuroinflammation and anxiety-like behavior in a rat model of myocardial infarction. Brain Behav..

[188] Luo K.W., Xia J., Cheng B.H., Gao H.C., Fu L.W., Luo X.L. (2020). Tea polyphenol EGCG inhibited colorectal-cancer-cell proliferation and migration via downregulation of STAT3. Gastroenterol. Rep..

[189] Gonzalez Suarez N., Rodriguez Torres S., Ouanouki A., El Cheikh-Hussein L., Annabi B. (2021). EGCG Inhibits Adipose-Derived Mesenchymal Stem Cells Differentiation into Adipocytes and Prevents a STAT3-Mediated Paracrine Oncogenic Control of Triple-Negative Breast Cancer Cell Invasive Phenotype. Molecules.

[190] Tang S.N., Fu J., Shankar S., Srivastava R.K. (2012). EGCG enhances the therapeutic potential of gemcitabine and CP690550 by inhibiting STAT3 signaling pathway in human pancreatic cancer. PLoS ONE.

[191] Zhu B.H., Chen H.Y., Zhan W.H., Wang C.Y., Cai S.R., Wang Z., Zhang C.H., He Y.L. (2011). (-)-Epigallocatechin-3-gallate inhibits VEGF expression induced by IL-6 via Stat3 in gastric cancer. World J. Gastroenterol..

[192] Khan N., Mukhtar H. (2018). Tea Polyphenols in Promotion of Human Health. Nutrients.

[193] Romano A., Martel F. (2021). The Role of EGCG in Breast Cancer Prevention and Therapy. Mini Rev. Med. Chem..

[194] Pervin M., Unno K., Ohishi T., Tanabe H., Miyoshi N., Nakamura Y. (2018). Beneficial Effects of Green Tea Catechins on Neurodegenerative Diseases. Molecules.

[195] Unno K., Pervin M., Taguchi K., Konishi T., Nakamura Y. (2020). Green Tea Catechins Trigger Immediate-Early Genes in the Hippocampus and Prevent Cognitive Decline and Lifespan Shortening. Molecules.

[196] Eng Q.Y., Thanikachalam P.V., Ramamurthy S. (2018). Molecular understanding of Epigallocatechin gallate (EGCG) in cardiovascular and metabolic diseases. J. Ethnopharmacol..

[197] Yamagata K. (2019). Polyphenols Regulate Endothelial Functions and Reduce the Risk of Cardiovascular Disease. Curr. Pharm. Des..

[198] Mokra D., Adamcakova J., Mokry J. (2022). Green Tea Polyphenol (-)-Epigallocatechin-3-Gallate (EGCG): A Time for a New Player in the Treatment of Respiratory Diseases?. Antioxidants.

[199] Carrasco-Pozo C., Cires M.J., Gotteland M. (2019). Quercetin and Epigallocatechin Gallate in the Prevention and Treatment of Obesity: From Molecular to Clinical Studies. J. Med. Food.

[200] Potenza M.A., Iacobazzi D., Sgarra L., Montagnani M. (2020). The Intrinsic Virtues of EGCG, an Extremely Good Cell Guardian, on Prevention and Treatment of Diabesity Complications. Molecules.

[201] Shahwan M., Alhumaydhi F., Ashraf G.M., Hasan P.M.Z., Shamsi A. (2022). Role of polyphenols in combating Type 2 Diabetes and insulin resistance. Int. J. Biol. Macromol..

[202] Cháirez-Ramírez M.H., de la Cruz-López K.G., García-Carrancá A. (2021). Polyphenols as Antitumor Agents Targeting Key Players in Cancer-Driving Signaling Pathways. Front. Pharmacol..

[203] Sigler K., Ruch R.J. (1993). Enhancement of gap junctional intercellular communication in tumor promoter-treated cells by components of green tea. Cancer Lett..

[204] Fan Y., Mao R., Yang J. (2013). NF-κB and STAT3 signaling pathways collaboratively link inflammation to cancer. Protein Cell.

[205] Afaq F., Adhami V.M., Ahmad N., Mukhtar H. (2003). Inhibition of ultraviolet B-mediated activation of nuclear factor κB in normal human epidermal keratinocytes by green tea constituent (−)-epigallocatechin-3-gallate. Oncogene.

[206] Gupta S., Hastak K., Afaq F., Ahmad N., Mukhtar H. (2004). Essential role of caspases in epigallocatechin-3-gallate-mediated inhibition of nuclear factor κB and induction of apoptosis. Oncogene.

[207] Adhami V.M., Siddiqui I.A., Ahmad N., Gupta S., Mukhtar H. (2004). Oral consumption of green tea polyphenols inhibits insulin-like growth factor-I-induced signaling in an autochthonous mouse model of prostate cancer. Cancer Res..

[208] Aggarwal B.B., Vijayalekshmi R.V., Sung B. (2009). Targeting inflammatory pathways for prevention and therapy of cancer: Short-term friend, long-term foe. Clin. Cancer Res..

[209] Nam S., Smith D.M., Dou Q.P. (2001). Ester bond-containing tea polyphenols potently inhibit proteasome activity in vitro and in vivo. J. Biol. Chem..

[210] Frei B., Higdon J.V. (2003). Antioxidant activity of tea polyphenols in vivo: Evidence from animal studies. J. Nutr..

[211] Yang G.Z., Wang Z.J., Bai F., Qin X.J., Cao J., Lv J.Y., Zhang M.S. (2015). Epigallocatechin-3-gallate protects HUVECs from PM2.5-induced oxidative stress injury by activating critical antioxidant pathways. Molecules.

[212] Ahmad N., Feyes D.K., Nieminen A.L., Agarwal R., Mukhtar H. (1997). Green tea constituent epigallocatechin-3-gallate and induction of apoptosis and cell cycle arrest in human carcinoma cells. J. Natl. Cancer Inst..

[213] Yang C.S., Maliakal P., Meng X. (2002). Inhibition of carcinogenesis by tea. Annu. Rev. Pharmacol. Toxicol..

[214] Sadava D., Whitlock E., Kane S.E. (2007). The green tea polyphenol, epigallocatechin-3-gallate inhibits telomerase and induces apoptosis in drug-resistant lung cancer cells. Biochem. Biophys. Res. Commun..

[215] Liu L., Zuo J., Wang G. (2017). Epigallocatechin-3-gallate suppresses cell proliferation and promotes apoptosis in Ec9706 and Eca109 esophageal carcinoma cells. Oncol. Lett..

[216] Cao Y., Cao R. (1999). Angiogenesis inhibited by drinking tea. Nature.

[217] Liu L., Lai C.Q., Nie L., Ordovas J., Band M., Moser L., Meydani M. (2008). The modulation of endothelial cell gene expression by green tea polyphenol-EGCG. Mol. Nutr. Food Res..

[218] Ahn W.S., Yoo J., Huh S.W., Kim C.K., Lee J.M., Namkoong S.E., Bae S.M., Lee I.P. (2003). Protective effects of green tea extract (polyphenon E and EGCG) on human cervical lesions. Eur. J. Cancer Prev..

[219] Jatoi A., Ellison N., Burch P.A., Sloan J.A., Dakhil S.R., Novotny P., Tan W., Fitch T.R., Rowland K.M., Young C.Y. (2003). A phase II trial of green tea in the treatment of patients with androgen independent metastatic prostate carcinoma. Cancer.

[220] Tsao A.S., Liu D., Martin J., Tang X.M., Lee J.J., El-Naggar A.K., Wistuba I., Culotta K.S., Mao L., Gillenwater A. (2009). Phase II randomized, placebo-controlled trial of green tea extract in patients with high-risk oral premalignant lesions. Cancer Prev. Res..

[221] Fujiki H., Sueoka E., Watanabe T., Suganuma M. (2015). Primary cancer prevention by green tea, and tertiary cancer prevention by the combination of green tea catechins and anticancer compounds. J. Cancer Prev..

[222] Kumar N.B., Pow-Sang J., Egan K.M., Spiess P.E., Dickinson S., Salup R., Helal M., McLarty J., Williams C.R., Schreiber F. (2015). Randomized, Placebo-Controlled Trial of Green Tea Catechins for Prostate Cancer Prevention. Cancer Prev. Res..

[223] Kumar N.B., Pow-Sang J., Spiess P.E., Park J., Salup R., Williams C.R., Parnes H., Schell M.J. (2016). Randomized, placebo-controlled trial evaluating the safety of one-year administration of green tea catechins. Oncotarget.

[224] Gee J.R., Saltzstein D.R., Kim K., Kolesar J., Huang W., Havighurst T.C., Wollmer B.W., Stublaski J., Downs T., Mukhtar H. (2017). A Phase II Randomized, Double-blind, Presurgical Trial of Polyphenon E in Bladder Cancer Patients to Evaluate Pharmacodynamics and Bladder Tissue Biomarkers. Cancer Prev. Res..

[225] Kiselev V.I., Ashrafyan L.A., Muyzhnek E.L., Gerfanova E.V., Antonova I.B., Aleshikova O.I., Sarkar F.H. (2018). A new promising way of maintenance therapy in advanced ovarian cancer: A comparative clinical study. BMC Cancer.

[226] Pervin M., Unno K., Takagaki A., Isemura M., Nakamura Y. (2019). Function of Green Tea Catechins in the Brain: Epigallocatechin Gallate and its Metabolites. Int. J. Mol. Sci..

[227] Sebastiani G., Almeida-Toledano L., Serra-Delgado M., Navarro-Tapia E., Sailer S., Valverde O., Garcia-Algar O., Andreu-Fernández V. (2021). Therapeutic Effects of Catechins in Less Common Neurological and Neurodegenerative Disorders. Nutrients.

[228] Wang Y., Wu S., Li Q., Lang W., Li W., Jiang X., Wan Z., Chen J., Wang H. (2022). Epigallocatechin-3-gallate: A phytochemical as a promising drug candidate for the treatment of Parkinson’s disease. Front. Pharmacol..

[229] Kuriyama S., Hozawa A., Ohmori K., Shimazu T., Matsui T., Ebihara S., Awata S., Nagatomi R., Arai H., Tsuji I. (2006). Green tea consumption and cognitive function: A cross-sectional study from the Tsurugaya Project 1. Am. J. Clin. Nutr..

[230] Kuriyama S., Shimazu T., Ohmori K., Kikuchi N., Nakaya N., Nishino Y., Tsubono Y., Tsuji I. (2006). Green tea consumption and mortality due to cardiovascular disease, cancer, and all causes in Japan: The Ohsaki study. JAMA.

[231] Feng L., Gwee X., Kua E.H., Ng T.P. (2010). Cognitive function and tea consumption in community dwelling older Chinese in Singapore. J. Nutr. Health Aging.

[232] Ide K., Yamada H., Takuma N., Park M., Wakamiya N., Nakase J., Ukawa Y., Sagesaka Y.M. (2014). Green tea consumption affects cognitive dysfunction in the elderly: A pilot study. Nutrients.

[233] Noguchi-Shinohara M., Yuki S., Dohmoto C., Ikeda Y., Samuraki M., Iwasa K., Yokogawa M., Asai K., Komai K., Nakamura H. (2014). Consumption of green tea, but not black tea or coffee, is associated with reduced risk of cognitive decline. PLoS ONE.

[234] Gu Y.J., He C.H., Li S., Zhang S.Y., Duan S.Y., Sun H.P., Shen Y.P., Xu Y., Yin J.Y., Pan C.W. (2018). Tea consumption is associated with cognitive impairment in older Chinese adults. Aging Ment. Health.

[235] Chang X., Rong C., Chen Y., Yang C., Hu Q., Mo Y., Zhang C., Gu X., Zhang L., He W. (2015). (-)-Epigallocatechin-3-gallate attenuates cognitive deterioration in Alzheimer’s disease model mice by upregulating neprilysin expression. Exp. Cell Res..

[236] Lee J.W., Lee Y.K., Ban J.O., Ha T.Y., Yun Y.P., Han S.B., Oh K.W., Hong J.T. (2009). Green tea (-)-epigallocatechin-3-gallate inhibits beta-amyloid-induced cognitive dysfunction through modification of secretase activity via inhibition of ERK and NF-kappaB pathways in mice. J. Nutr..

[237] Yamamoto N., Shibata M., Ishikuro R., Tanida M., Taniguchi Y., Ikeda-Matsuo Y., Sobue K. (2017). Epigallocatechin gallate induces extracellular degradation of amyloid β-protein by increasing neprilysin secretion from astrocytes through activation of ERK and PI3K pathways. Neuroscience.

[238] Guo Y., Zhao Y., Nan Y., Wang X., Chen Y., Wang S. (2017). (-)-Epigallocatechin-3-gallate ameliorates memory impairment and rescues the abnormal synaptic protein levels in the frontal cortex and hippocampus in a mouse model of Alzheimer’s disease. Neuroreport.

[239] Nan S., Wang P., Zhang Y., Fan J. (2021). Epigallocatechin-3-Gallate Provides Protection Against Alzheimer’s Disease-Induced Learning and Memory Impairments in Rats. Drug Des. Devel. Ther..

[240] Barranco Quintana J.L., Allam M.F., Del Castillo A.S., Navajas R.F. (2009). Parkinson’s disease and tea: A quantitative review. J. Am. Coll. Nutr..

[241] Hosseini Tabatabaei N., Babakhani B., Hosseini Tabatabaei A., Vahabi Z., Soltanzadeh A. (2013). Non-genetic factors associated with the risk of Parkinson’s disease in Iranian patients. Funct. Neurol..

[242] Tanaka K., Miyake Y., Fukushima W., Sasaki S., Kiyohara C., Tsuboi Y., Yamada T., Oeda T., Miki T., Kawamura N. (2011). Intake of Japanese and Chinese teas reduces risk of Parkinson’s disease. Park. Relat. Disord..

[243] Singh N.A., Mandal A.K., Khan Z.A. (2016). Potential neuroprotective properties of epigallocatechin-3-gallate (EGCG). Nutr. J..

[244] Xu Y., Zhang Y., Quan Z., Wong W., Guo J., Zhang R., Yang Q., Dai R., McGeer P.L., Qing H. (2016). Epigallocatechin Gallate (EGCG) Inhibits Alpha-Synuclein Aggregation: A Potential Agent for Parkinson’s Disease. Neurochem. Res..

[245] Levites Y., Weinreb O., Maor G., Youdim M.B., Mandel S. (2001). Green tea polyphenol (-)-epigallocatechin-3-gallate prevents N-methyl-4-phenyl-1,2,3,6-tetrahydropyridine-induced dopaminergic neurodegeneration. J. Neurochem..

[246] Xu Q., Langley M., Kanthasamy A.G., Reddy M.B. (2017). Epigallocatechin Gallate Has a Neurorescue Effect in a Mouse Model of Parkinson Disease. J. Nutr..

[247] Kim J.S., Kim J.M., O J.J., Jeon B.S. (2010). Inhibition of inducible nitric oxide synthase expression and cell death by (-)-epigallocatechin-3-gallate, a green tea catechin, in the 1-methyl-4-phenyl-1,2,3,6-tetrahydropyridine mouse model of Parkinson’s disease. J. Clin. Neurosci..

[248] Stensvold I., Tverdal A., Solvoll K., Foss O.P. (1992). Tea consumption. relationship to cholesterol, blood pressure, and coronary and total mortality. Prev. Med..

[249] Xu X., Pan J., Zhou X. (2014). Amelioration of lipid profile and level of antioxidant activities by epigallocatechin-gallate in a rat model of atherogenesis. Heart Lung Circ..

[250] Li J., Ye L., Wang X., Liu J., Wang Y., Zhou Y., Ho W. (2012). (-)-Epigallocatechin gallate inhibits endotoxin-induced expression of inflammatory cytokines in human cerebral microvascular endothelial cells. J. Neuroinflammation..

[251] Liao Z.L., Zeng B.H., Wang W., Li G.H., Wu F., Wang L., Zhong Q.P., Wei H., Fang X. (2016). Impact of the Consumption of Tea Polyphenols on Early Atherosclerotic Lesion Formation and Intestinal Bifidobacteria in High-Fat-Fed ApoE-/- Mice. Front. Nutr..

[252] Widlansky M.E., Hamburg N.M., Anter E., Holbrook M., Kahn D.F., Elliott J.G., Keaney J.F., Vita J.A. (2007). Acute EGCG supplementation reverses endothelial dysfunction in patients with coronary artery disease. J. Am. Coll. Nutr..

[253] Anter E., Chen K., Shapira O.M., Karas R.H., Keaney J.F. (2005). p38 mitogen-activated protein kinase activates eNOS in endothelial cells by an estrogen receptor alpha-dependent pathway in response to black tea polyphenols. Circ. Res..

[254] Barton M., Haudenschild C.C., d’Uscio L.V., Shaw S., Munter K., Luscher T.F. (1998). Endothelin ETA receptor blockade restores NO-mediated endothelial function and inhibits atherosclerosis in apolipoprotein E-deficient mice. Proc. Natl. Acad. Sci. USA.

[255] Reiter C.E., Kim J.A., Quon M.J. (2010). Green tea polyphenol epigallocatechin gallate reduces endothelin-1 expression and secretion in vascular endothelial cells: Roles for AMP-activated protein kinase, Akt, and FOXO1. Endocrinology.

[256] Moyle C.W., Cerezo A.B., Winterbone M.S., Hollands W.J., Alexeev Y., Needs P.W., Kroon P.A. (2015). Potent inhibition of VEGFR-2 activation by tight binding of green tea epigallocatechin gallate and apple procyanidins to VEGF: Relevance to angiogenesis. Mol. Nutr. Food Res..

[257] Kang W.S., Chung K.H., Chung J.H., Lee J.Y., Park J.B., Zhang Y.H., Yoo H.S., Yun Y.P. (2001). Antiplatelet activity of green tea catechins is mediated by inhibition of cytoplasmic calcium increase. J. Cardiovasc. Pharmacol..

[258] Jin Y.R., Im J.H., Park E.S., Cho M.R., Han X.H., Lee J.J., Lim Y., Kim T.J., Yun Y.P. (2008). Antiplatelet activity of epigallocatechin gallate is mediated by the inhibition of PLCgamma2 phosphorylation, elevation of PGD2 production, and maintaining calcium-ATPase activity. J. Cardiovasc. Pharmacol..

[259] Shenouda S.M., Vita J.A. (2007). Effects of flavonoid-containing beverages and EGCG on endothelial function. J. Am. Coll. Nutr..

[260] Wang M., Zhong H., Zhang X., Huang X., Wang J., Li Z., Chen M., Xiao Z. (2021). EGCG promotes PRKCA expression to alleviate LPS-induced acute lung injury and inflammatory response. Sci. Rep..

[261] Almatroodi S.A., Almatroudi A., Alsahli M.A., Aljasir M.A., Syed M.A., Rahmani A.H. (2020). Epigallocatechin-3-Gallate (EGCG), an Active Compound of Green Tea Attenuates Acute Lung Injury Regulating Macrophage Polarization and Krüpple-Like-Factor 4 (KLF4) Expression. Molecules.

[262] Tang H., Hao S., Khan M.F., Zhao L., Shi F., Li Y., Guo H., Zou Y., Lv C., Luo J. (2022). Epigallocatechin-3-Gallate Ameliorates Acute Lung Damage by Inhibiting Quorum-Sensing-Related Virulence Factors of Pseudomonas aeruginosa. Front. Microbiol..

[263] Sharma A., Vaghasiya K., Ray E., Gupta P., Gupta U.D., Singh A.K., Verma R.K. (2020). Targeted Pulmonary Delivery of the Green Tea Polyphenol Epigallocatechin Gallate Controls the Growth of Mycobacterium tuberculosis by Enhancing the Autophagy and Suppressing Bacterial Burden. ACS Biomater. Sci. Eng..

[264] Ling J.X., Wei F., Li N., Li J.L., Chen L.J., Liu Y.Y., Luo F., Xiong H.R., Hou W., Yang Z.Q. (2012). Amelioration of influenza virus-induced reactive oxygen species formation by epigallocatechin gallate derived from green tea. Acta Pharmacol. Sin..

[265] Henss L., Auste A., Schürmann C., Schmidt C., von Rhein C., Mühlebach M.D., Schnierle B.S. (2021). The green tea catechin epigallocatechin gallate inhibits SARS-CoV-2 infection. J. Gen. Virol..

[266] Liu J., Bodnar B.H., Meng F., Khan A.I., Wang X., Saribas S., Wang T., Lohani S.C., Wang P., Wei Z. (2021). Epigallocatechin gallate from green tea effectively blocks infection of SARS-CoV-2 and new variants by inhibiting spike binding to ACE2 receptor. Cell Biosci..

[267] Du A., Zheng R., Disoma C., Li S., Chen Z., Li S., Liu P., Zhou Y., Shen Y., Liu S. (2021). Epigallocatechin-3-gallate, an active ingredient of Traditional Chinese Medicines, inhibits the 3CLpro activity of SARS-CoV-2. Int. J. Biol. Macromol..

[268] Jang M., Park R., Park Y.I., Cha Y.E., Yamamoto A., Lee J.I., Park J. (2021). EGCG, a green tea polyphenol, inhibits human coronavirus replication in vitro. Biochem. Biophys. Res. Commun..

[269] Park R., Jang M., Park Y.I., Park Y., Jung W., Park J., Park J. (2021). Epigallocatechin Gallate (EGCG), a Green Tea Polyphenol, Reduces Coronavirus Replication in a Mouse Model. Viruses.

[270] Laforge M., Elbim C., Frère C., Hémadi M., Massaad C., Nuss P., Benoliel J.J., Becker C. (2020). Tissue damage from neutrophil-induced oxidative stress in COVID-19. Nat. Rev. Immunol..

[271] Wan Q., Song D., Li H., He M.L. (2020). Stress proteins: The biological functions in virus infection, present and challenges for target-based antiviral drug development. Signal Transduct. Target. Ther..

[272] Li W., Zhu S., Li J., Assa A., Jundoria A., Xu J., Fan S., Eissa N.T., Tracey K.J., Sama A.E. (2011). EGCG stimulates autophagy and reduces cytoplasmic HMGB1 levels in endotoxin-stimulated macrophages. Biochem. Pharmacol..

[273] Lu B., Antoine D.J., Kwan K., Lundbäck P., Wähämaa H., Schierbeck H., Robinson M., Van Zoelen M.A., Yang H., Li J. (2014). JAK/STAT1 signaling promotes HMGB1 hyperacetylation and nuclear translocation. Proc. Natl. Acad. Sci. USA.

[274] Holy E.W., Stämpfli S.F., Akhmedov A., Holm N., Camici G.G., Lüscher T.F., Tanner F.C. (2010). Laminin receptor activation inhibits endothelial tissue factor expression. J. Mol. Cell. Cardiol..

[275] Choi Y.S., Bae C.H., Song S.Y., Kim Y.D. (2014). The effect of Epigallocatechin-3-gallate in allergic airway inflammation. Rhinology..

[276] Shan L., Kang X., Liu F., Cai X., Han X., Shang Y. (2018). Epigallocatechin gallate improves airway inflammation through TGF-β1 signaling pathway in asthmatic mice. Mol. Med. Rep..

[277] André D.M., Horimoto C.M., Calixto M.C., Alexandre E.C., Antunes E. (2018). Epigallocatechin-3-gallate protects against the exacerbation of allergic eosinophilic inflammation associated with obesity in mice. Int. Immunopharmacol..

[278] Kim S.H., Park H.J., Lee C.M., Choi I.W., Moon D.O., Roh H.J., Lee H.K., Park Y.M. (2006). Epigallocatechin-3-gallate protects toluene diisocyanate-induced airway inflammation in a murine model of asthma. FEBS Lett..

[279] Li Y., Chen L., Guo F., Cao Y., Hu W., Shi Y., Lin X., Hou J., Li L., Ding X. (2019). Effects of epigallocatechin-3-gallate on the HMGB1/RAGE pathway in PM2.5-exposed asthmatic rats. Biochem. Biophys. Res. Commun..

[280] Yang N., Li X. (2022). Epigallocatechin gallate relieves asthmatic symptoms in mice by suppressing HIF-1α/VEGFA-mediated M2 skewing of macrophages. Biochem. Pharmacol..

[281] Liang Y., Liu K.W.K., Yeung S.C., Li X., Ip M.S.M., Mak J.C.W. (2017). (-)-Epigallocatechin-3-gallate Reduces Cigarette Smoke-Induced Airway Neutrophilic Inflammation and Mucin Hypersecretion in Rats. Front. Pharmacol..

[282] Wu B., Sodji Q.H., Oyelere A.K. (2022). Inflammation, Fibrosis and Cancer: Mechanisms, Therapeutic Options and Challenges. Cancers.

[283] Sriram N., Kalayarasan S., Sudhandiran G. (2008). Enhancement of antioxidant defense system by epigallocatechin-3-gallate during bleomycin induced experimental pulmonary fibrosis. Biol. Pharm. Bull..

[284] Sriram N., Kalayarasan S., Sudhandiran G. (2009). Epigallocatechin-3-gallate augments antioxidant activities and inhibits inflammation during bleomycin-induced experimental pulmonary fibrosis through Nrf2-Keap1 signaling. Pulm. Pharmacol. Ther..

[285] Sriram N., Kalayarasan S., Sudhandiran G. (2009). Epigallocatechin-3-gallate exhibits anti-fibrotic effect by attenuating bleomycin-induced glycoconjugates, lysosomal hydrolases and ultrastructural changes in rat model pulmonary fibrosis. Chem. Biol. Interact..

[286] Sriram N., Kalayarasan S., Manikandan R., Arumugam M., Sudhandiran G. (2015). Epigallocatechin gallate attenuates fibroblast proliferation and excessive collagen production by effectively intervening TGF-β1 signalling. Clin. Exp. Pharmacol. Physiol..

[287] Wei Y., Dong W., Jackson J., Ho T.C., Le Saux C.J., Brumwell A., Li X., Klesney-Tait J., Cohen M.L., Wolters P.J. (2021). Blocking LOXL2 and TGFβ1 signalling induces collagen I turnover in precision-cut lung slices derived from patients with idiopathic pulmonary fibrosis. Thorax.

[288] Hamdy M.A., El-Maraghy S.A., Kortam M.A. (2012). Modulatory effects of curcumin and green tea extract against experimentally induced pulmonary fibrosis: A comparison with N-acetyl cysteine. J. Biochem. Mol. Toxicol..

[289] Kim H.R., Park B.K., Oh Y.M., Lee Y.S., Lee D.S., Kim H.K., Kim J.Y., Shim T.S., Lee S.D. (2006). Green tea extract inhibits paraquat-induced pulmonary fibrosis by suppression of oxidative stress and endothelin-l expression. Lung.

[290] Yao J.-J., Ma Q.-Q., Shen W.-W., Li L.-C., Hu D. (2022). Nano-enabled delivery of EGCG ameliorates silica-induced pulmonary fibrosis in rats. Toxicology.

[291] Wolfram S., Wang Y., Thielecke F. (2006). Anti-obesity effects of green tea: From bedside to bench. Mol. Nutr. Food Res..

[292] Sae-Tan S., Grove K.A., Lambert J.D. (2011). Weight control and prevention of metabolic syndrome by green tea. Pharmacol. Res..

[293] Bose M., Lambert J.D., Ju J., Reuhl K.R., Shapses S.A., Yang C.S. (2008). The major green tea polyphenol, (-)-epigallocatechin-3-gallate, inhibits obesity, metabolic syndrome, and fatty liver disease in high-fat-fed mice. J. Nutr..

[294] Xin X., Cheng C., Bei-Yu C., Hong-Shan L., Hua-Jie T., Xin W., Zi-Ming A., Qin-Mei S., Yi-Yang H., Qin F. (2021). Caffeine and EGCG Alleviate High-Trans Fatty Acid and High-Carbohydrate Diet-Induced NASH in Mice: Commonality and Specificity. Front. Nutr..

[295] Chen Y.K., Cheung C., Reuhl K.R., Liu A.B., Lee M.J., Lu Y.P., Yang C.S. (2011). Effects of green tea polyphenol (-)-epigallocatechin-3-gallate on newly developed high-fat/Western-style diet-induced obesity and metabolic syndrome in mice. J. Agric. Food Chem..

[296] Sae-Tan S., Grove K.A., Kennett M.J., Lambert J.D. (2011). (-)-Epigallocatechin-3-gallate increases the expression of genes related to fat oxidation in the skeletal muscle of high fat-fed mice. Food Funct..

[297] Huang J., Feng S., Liu A., Dai Z., Wang H., Reuhl K., Lu W., Yang C.S. (2018). Green Tea Polyphenol EGCG Alleviates Metabolic Abnormality and Fatty Liver by Decreasing Bile Acid and Lipid Absorption in Mice. Mol. Nutr. Food Res..

[298] Chatree S., Sitticharoon C., Maikaew P., Pongwattanapakin K., Keadkraichaiwat I., Churintaraphan M., Sripong C., Sririwichitchai R., Tapechum S. (2021). Epigallocatechin gallate decreases plasma triglyceride, blood pressure, and serum kisspeptin in obese human subjects. Exp. Biol. Med..

[299] Huang L.H., Liu C.Y., Wang L.Y., Huang C.J., Hsu C.H. (2018). Effects of green tea extract on overweight and obese women with high levels of low density-lipoprotein-cholesterol (LDL-C): A randomised, double-blind, and cross-over placebo-controlled clinical trial. BMC Complement. Altern. Med..

[300] Chen I.J., Liu C.Y., Chiu J.P., Hsu C.H. (2016). Therapeutic effect of high-dose green tea extract on weight reduction: A randomized, double-blind, placebo-controlled clinical trial. Clin. Nutr..

[301] Kim Y., Keogh J.B., Clifton P.M. (2016). Polyphenols and Glycemic Control. Nutrients.

[302] Zhang C., Li X., Hu X., Xu Q., Zhang Y., Liu H., Diao Y., Zhang X., Li L., Yu J. (2021). Epigallocatechin-3-gallate prevents inflammation and diabetes -Induced glucose tolerance through inhibition of NLRP3 inflammasome activation. Int. Immunopharmacol..

[303] Van Woudenbergh G.J., Kuijsten A., Drogan D., van der A D.L., Romaguera D., Ardanaz E., Amiano P., Barricarte A., Beulens J.W., Boeing H. (2012). Tea consumption and incidence of type 2 diabetes in Europe: The EPIC-InterAct case-cohort study. PLoS ONE.

[304] Hamer M., Witte D.R., Mosdøl A., Marmot M.G., Brunner E.J. (2008). Prospective study of coffee and tea consumption in relation to risk of type 2 diabetes mellitus among men and women: The Whitehall II study. Br. J. Nutr..

[305] Montagnani M., Golovchenko I., Kim I., Koh G.Y., Goalstone M.L., Mundhekar A.N., Johansen M., Kucik D.F., Quon M.J., Draznin B. (2002). Inhibition of phosphatidylinositol 3-kinase enhances mitogenic actions of insulin in endothelial cells. J. Biol. Chem..

[306] Kim J.A., Montagnani M., Koh K.K., Quon M.J. (2006). Reciprocal relationships between insulin resistance and endothelial dysfunction: Molecular and pathophysiological mechanisms. Circulation.

[307] Yang J., Han Y., Chen C., Sun H., He D., Guo J., Jiang B., Zhou L., Zeng C. (2013). EGCG attenuates high glucose-induced endothelial cell inflammation by suppression of PKC and NF-κB signaling in human umbilical vein endothelial cells. Life Sci..

